# Drug target gene-based analyses of drug repositionability in rare and intractable diseases

**DOI:** 10.1038/s41598-021-91428-4

**Published:** 2021-06-11

**Authors:** Ryuichi Sakate, Tomonori Kimura

**Affiliations:** 1 Laboratory of Rare Disease Resource Library, Center for Rare Disease Research, National Institutes of Biomedical Innovation, Health and Nutrition (NIBIOHN), Tokyo, Japan; 2 Platform of Therapeutics for Rare Disease, Center for Rare Disease Research, National Institutes of Biomedical Innovation, Health and Nutrition (NIBIOHN), Tokyo, Japan; 3 Reverse Translational Research Project, Center for Rare Disease Research, National Institutes of Biomedical Innovation, Health and Nutrition (NIBIOHN), Tokyo, Japan; 4 KAGAMI Project, National Institutes of Biomedical Innovation, Health and Nutrition (NIBIOHN), Tokyo, Japan

**Keywords:** Computational biology and bioinformatics, Drug discovery, Diseases, Medical research

## Abstract

Drug development for rare and intractable diseases has been challenging for decades due to the low prevalence and insufficient information on these diseases. Drug repositioning is increasingly being used as a promising option in drug development. We aimed to analyze the trend of drug repositioning and inter-disease drug repositionability among rare and intractable diseases. We created a list of rare and intractable diseases based on the designated diseases in Japan. Drug information extracted from clinical trial data were integrated with information of drug target genes, which represent the mechanism of drug action. We obtained 753 drugs and 551 drug target genes from 8307 clinical trials for 189 diseases or disease groups. Trend analysis of drug sharing between a disease pair revealed that 1676 drug repositioning events occurred in 4401 disease pairs. A score, *R*_*gene*,_ was invented to investigate the proportion of drug target genes shared between a disease pair. Annual changes of *R*_*gene*_ corresponded to the trend of drug repositioning and predicted drug repositioning events occurring within a year or two. Drug target gene-based analyses well visualized the drug repositioning landscape. This approach facilitates drug development for rare and intractable diseases.

## Introduction

Rare and intractable diseases are mostly severe and cause a lifetime suffering^1^. There are approximately 7000 rare diseases and more than 300 million people are affected worldwide^2,3^. Because of their low prevalence and complex pathology, they are often difficult to diagnose and treat. A paradigm shift and systematic approach to drug discovery is the key for treatment of rare and intractable diseases.

Recently, drug development in rare and intractable diseases has attracted the attention of pharmaceutical companies. New modalities, such as antibodies and anti-sense oligonucleotides, are being developed as an effective approach to these diseases. Another solution is drug repositioning^4,5^. Drug repositioning, also called drug repurposing, introduces existing drugs in the treatment of new diseases, where their use has not been previously tested. By utilizing drugs already approved as safe in previous clinical trials, drug repositioning can accelerate drug discovery also in rare and intractable diseases.

The trend of drug discovery and repositioning in rare and intractable diseases has been unclear. The main reason for this is the lack of comprehensive information for the diseases. Apart from the small number of patients, the variable classification, challenging diagnosis, and the use of inconsistent disease names make the integration of information particularly difficult. Investigation of information is crucial also for accelerating drug repositioning in rare and intractable diseases.

When repositioning a drug developed for a specific disease, it is important to find a target disease that has a common mechanism of action for the particular drug^6–8^. We considered that drug target genes are able to provide key information for the mechanism. Each drug has a set of target genes (or target gene products), with which the drug interacts to modify their actions. Sharing the target genes of a drug between a disease pair may suggest that this disease pair also shares a common mechanism of drug action. Therefore, a disease pair sharing drug target genes could be a good candidate for drug repositioning.

In this study, we collected drug information from four major clinical trial registries, and assigned information of target genes to these drugs. We targeted 333 rare and intractable diseases, which consists of 787 main and sub-categorized diseases in total. We hypothesized that a proportion of the number of shared genes to that of total genes for each disease in the pair could be the key for drug repositionability. Through the inter-disease analysis based on drug target genes, we invented a score of drug repositionability that is useful for drug development in rare and intractable diseases.

## Results

### Trend of drug development for rare and intractable diseases

To visualize the inter-disease landscape of drug repositioning in rare and intractable diseases, we extracted information on drug development from clinical trial registries. Comprehensive information on drug development is available from clinical trial registries, since it is mandatory to register all clinical trials in at least one registry worldwide. Then, we investigated genes or gene products that are targeted by the drugs. These target genes, which can represent the mechanism of drug action, were used for mining the drug repositionability in this study.

In order to search clinical trials, we constructed an original list of rare and intractable diseases based on the intractable diseases designated in Japan (Supplementary Table 1). This disease list included synonyms and consisted of 787 rare and intractable diseases in a two-level categorized format (333 first-level diseases and 454 second-level diseases). Since the number of drugs for rare and intractable diseases is mostly small, we performed the following analyses based on the 333 first-level diseases, by merging all search results for the second-level diseases to their parent (first-level) diseases. The disease list covered 15 disease fields, such as neuromuscular diseases, metabolic diseases, cutaneous connective tissue diseases, etc.^9^. We also constructed a reference drug list that included drug names, synonyms, and product names registered in DrugBank^10^. This drug list consisted of 13,339 drugs (DrugBank accession numbers) and was used for searching clinical trials.

First, by searching 412,555 clinical trials from the four largest registries in the world (JPRN^11^, ClinicalTrials.gov^12^, EU-CTR^13^, and ChiCTR^14^) through WHO International Clinical Trials Registry Platform^15^, 15,194 trials were identified to target rare and intractable diseases and contain drug information (Fig. 1A). In these trials, 1666 drugs were tested for 226 diseases in total. Second, we extracted clinical trials in which at least one drug had target gene information based on KEGG^16^. As a result, we selected 8307 clinical trials that tested 753 drugs which targeted 551 genes (Supplementary Table 2) for 189 rare and intractable diseases (Fig. 1A, Supplementary Table 3). Out of the 8307 clinical trials, 3241 trials (39.0%) were Phase 3 or 4 trials (Supplementary Fig. 1). We used these 8307 trials as a data set for the analysis, since the target gene information is a key to investigate inter-disease relationship that might underlie drug repositioning.

**Figure ﻿1:**
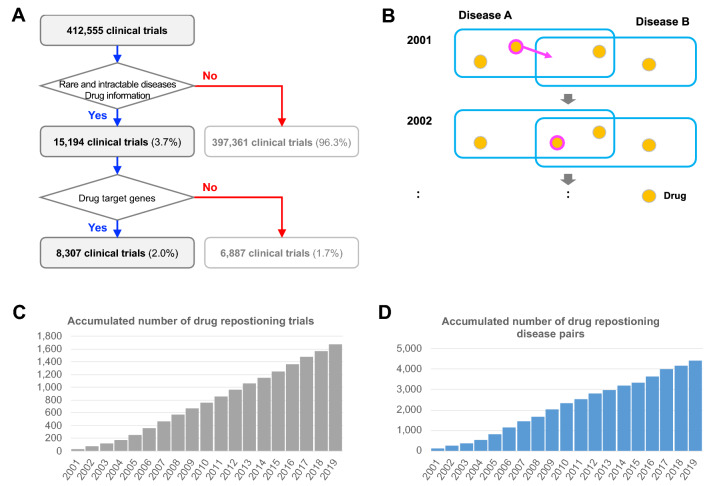
Analysis of drug repositioning in rare and intractable diseases based on clinical trial data. (**A**) Pipeline of extracting information from clinical trials. First, from 412,555 clinical trials in the four registries (JPRN, ClinicalTrials.gov, EU-CTR, and ChiCTR), 15,194 trials which are for rare and intractable diseases and contain drug information were selected. Second, by limiting the drugs to those that have target gene information, 8307 trials containing those drugs were selected. (**B**) Schema of drug repositioning where a drug in disease A is repositioned to disease B. A drug repositioning occurs when a drug which formerly tested for a disease newly becomes shared. (**C**) Accumulated number of clinical trials identified as drug repositioning trials enrolled annually in recent 20 years. In total, 1676 clinical trials were identified during the period. (**D**) Accumulated number of disease pairs combined for drug repositioning first appeared during the period. In total 4401 disease pairs were counted during the period.

### Drug repositioning in rare and intractable diseases

In the extracted clinical trials, 442 out of 753 drugs were tested for two or more diseases (Supplementary Fig. 2), suggesting that these drugs had been tested as repositioned drugs among rare and intractable diseases. Since drug repositioning is a process of finding another target disease, we examined the trend of drug repositioning from the viewpoint of a disease pair. Drug repositioning in a disease pair was defined as a drug donor (provider) and a drug recipient (acceptor), that is, a drug which is developed for one disease (drug donor) is repositioned for another disease (drug recipient, Fig. 1B). When repositioned, the drug becomes shared between the disease pair.

We defined a drug repositioning event as an annual change of drug sets tested in clinical trials between a disease pair. Clinical trials purposing drug repositioning were selected based on two criteria; (i) a drug was newly tested for a disease in the trial, and (ii) this drug had been already tested for other rare and intractable diseases in other trials. During the past 20 years, the number of clinical trials identified as drug repositioning trials continuously increased to 1676 trials in November 2019 (Fig. 1C). The unique number of disease pairs for drug repositioning also increased to 4401 pairs during the same period (Fig. 1D). As a whole, drug repositioning was actively occurring in rare and intractable diseases.

### Invention of drug target gene-based score for the analysis of drug repositioning landscape

**Figure 2:**
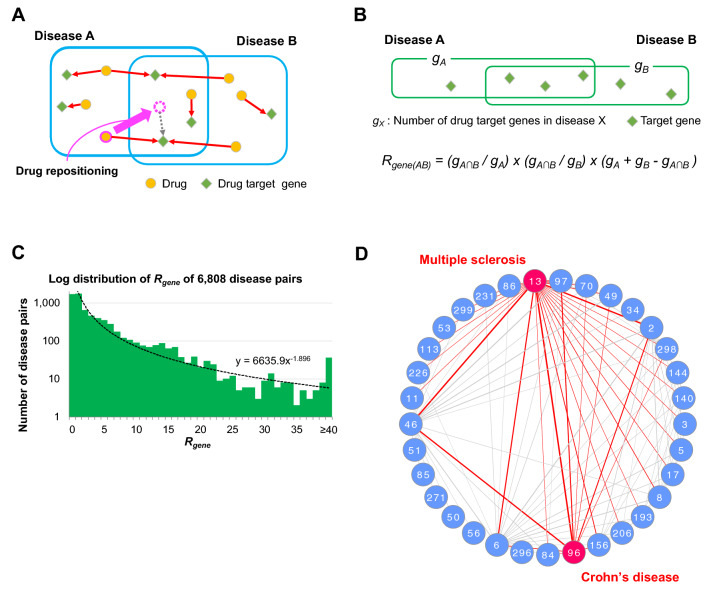
*R*_*gene*_: A score of drug repositionability based on sharedness of drug target genes between a disease pair. (**A**) Schema of drug repositioning where a drug in disease A is repositioned to disease B, with information of drug target genes. Drugs and their target genes are differently positioned across the three regions (disease A only, disease B only, and disease A and B). (**B**) Calculation of *R*_*gene*_ as a score of drug target gene sharedness between disease A and disease B. (**C**) Log distribution of *R*_*gene*_ of 6808 disease pairs. *R*_*gene*_ of 2466 disease pairs (36.2%) was less than 1. Note: disease pairs which had no repositioned drugs are included. (**D**) Inter-disease network by *R*_*gene*_. A circular network consists of 33 diseases from the top 100 *R*_*gene*_ disease pairs were depicted. The number of disease pairs (connected lines) are different among the diseases. Multiple sclerosis (ID:13) and Crohn’s disease (ID:96) are paired to many other diseases.

We investigated the drug repositionability among rare and intractable diseases based on drug target genes. Drug target genes can represent the inter-disease proximity more clearly than drugs, as denoting similar mechanisms of drug actions that lead to drug repositionability between diseases. We connected drugs found in the clinical trials to their target genes, then we examined whether the drug target genes for each disease became shared between diseases (Fig. 2A). Some drugs target more than one gene, while some genes are targeted by more than one drug.

To investigate the drug target gene-based proximity of a pair of diseases, we invented a score, *R*_*gene*_ (Relationship between diseases based on drug target gene sharing). *R*_*gene*_ is calculated for each disease pair by multiplying the proportions of the number of shared genes to the number of total genes for each disease, and the unique number of total genes for both diseases (Fig. 2B). A larger *R*_*gene*_ indicates a higher similarity in the mechanisms of drug actions between the disease pair. The distribution of *R*_*gene*_ was examined in 6808 disease pairs (Fig. 2C). This distribution ranged between 0.03 and 107.78, and was extremely biased; *R*_*gene*_ of 2466 disease pairs (36.2%) was less than 1, while only 814 pairs (12.0%) were higher than 10, and 220 pairs (3.2%) were higher than 20. Higher *R*_*gene*_ was limited to a small number of disease pairs.

The top 10 *R*_*gene*_ disease pairs are listed in Table 1. The diseases in this list were composed of a specific subset of diseases and disease fields. Neuromuscular diseases (Multiple sclerosis, amyotrophic lateral sclerosis, and Parkinson’s disease) and digestive diseases (Crohn’s disease and ulcerative colitis) appeared multiple times in the either side of disease pairs. Some disease pairs were paired by diseases from different disease fields as seen in the pair of Multiple sclerosis (neuromuscular diseases) and Crohn’s disease (digestive diseases).

**Table 1:** Top 10 *R*_*gene*_ disease pairs (A. Neuromuscular diseases, D. Immune diseases, M. Digestive diseases).

Rank	Disease A		Disease B		*R*_*gene*_
	ID	Name	ID	Name	
1	13	Multiple sclerosis [A]	96	Crohn’s disease [M]	107.78
2	13	Multiple sclerosis [A]	46	Malignant rheumatoid arthritis [D]	103.88
3	2	Amyotrophic lateral sclerosis [A]	13	Multiple sclerosis [A]	99.64
4	46	Malignant rheumatoid arthritis [D]	96	Crohn’s disease [M]	92.11
5	6	Parkinson’s disease [A]	13	Multiple sclerosis [A]	82.27
6	46	Malignant rheumatoid arthritis [D]	97	Ulcerative colitis [M]	75.84
7	96	Crohn’s disease [M]	97	Ulcerative colitis [M]	75.07
8	2	Amyotrophic lateral sclerosis [A]	96	Crohn’s disease [M]	72.50
9	2	Amyotrophic lateral sclerosis [A]	6	Parkinson’s disease [A]	69.91
10	13	Multiple sclerosis [A]	97	Ulcerative colitis [M]	66.94

This trend of biases was also observed in the following disease network analysis. We visualized the drug repositionability network by merging the top 100 *R*_*gene*_ disease pairs as a network (Fig. 2D). This network consisted of 33 diseases (Supplementary Table 5), since some diseases appeared more than once in the pairs as shown in Table 1. As expected, these diseases had multiple partners in the network. For example, multiple sclerosis (ID: 13) and Crohn’s disease (ID: 96) not only paired together, but also paired with many other diseases. The disease network based on drug target genes is expected to reveal a drug repositionability landscape as a wide variety of inter-disease relationships in rare and intractable diseases.

### ***R***_***gene***_ reflects and predicts drug repositioning events

**Figure 3:**
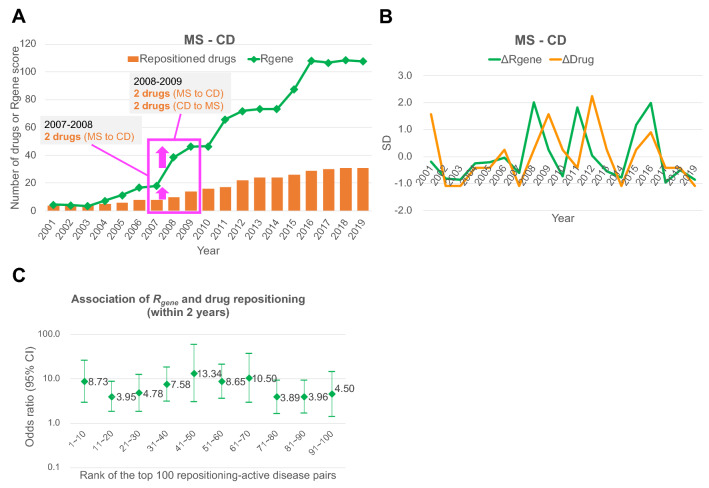
Annual changes of *R*_*gene*_ and drug repositioning between disease pairs in recent 20 years. (**A**) Annual changes of *R*_*gene*_ and drug repositioning between multiple sclerosis (MS) and Crohn’s disease (CD). *R*_*gene*_ increased gradually correlating to that of the number of repositioned drugs during the whole period. Three characteristic surges were seen for both *R*_*gene*_ and drug repositioning in 2007–2008, 2010–2011, and 2014–2016. (**B**) For MS and CD pair, annual changes of *R*_*gene*_ and drug repositioning were standardized by SD. The three surges of *R*_*gene*_ (*ΔR*_*gene*_) appeared to precede corresponding surges of drug repositioning (*ΔDrug*) by about a year. (**C**) Odds ratios of *R*_*gene*_ and drug repositioning as an explanatory variable and an objective variable, respectively, by logistic regression analyses. The top 100 repositioning-active disease pairs were divided into 10 groups consecutively and examined the predictability by *R*_*gene*_. This analysis validated the ubiquitous predictability by *R*_*gene*_. Note: In fact, 93 disease pairs were used since 7 pairs lacked drug repositioning events.

We investigated how *R*_*gene*_ represents drug repositioning events over the past 20 years. We calculated annual changes in *R*_*gene*_ and the number of repositioned drugs in each disease pair. Here, we selected multiple sclerosis (MS) and Crohn’s disease (CD) in Table 1, for instance. Between MS and CD, *R*_*gene*_ increased gradually during the period with three characteristic surges seen from 2007 to 2008, 2010 to 2011, and 2014 to 2016 (Fig. 3A). We found that these surges of *R*_*gene*_ coincided with actual drug repositioning events between MS and CD. In 2007–2008 when the first surge of *R*_*gene*_ was observed, two drugs (cholecalciferol and glatiramer) were repositioned from MS to CD. In the following 2008–2009, additionally, four drugs were repositioned (tacrolimus and dexamethasone from MS to CD; zoledronic acid and prednisolone from CD to MS). As described above, we found the tendency that *R*_*gene*_ surges coincided with, or preceded to, the actual drug repositioning events.

We investigated whether this tendency holds true in other disease pairs. For this purpose, we standardized the variations of *R*_*gene*_ and the number of repositioned drugs during the period. For each disease pair, annual changes in *R*_*gene*_ and the number of drugs were converted to *ΔR*_*gene*_ and *ΔDrug*, respectively, based on their standard deviations throughout the period (see Methods for details). As a result, *ΔR*_*gene*_ clearly visualizes three surges in *R*_*gene*_ in the MS–CD pair, which are followed by rapid increases of *ΔDrug* within 2 years (Fig. 3B). This tendency is comprehensively observed in other disease pairs in Table 1: Amyotrophic lateral sclerosis (ALS)–MS, Parkinson’s disease (PD)–MS, and ALS–PD (Supplementary Figs. 3 and 4). Through these observations of *ΔR*_*gene*_ and *ΔDrug*, we found that drug repositioning events surge periodically like a wave, and that *ΔR*_*gene*_ can suitably represent this drug repositioning wave.

The surge of *R*_*gene*_ is likely to precede drug repositioning events actively occurring. We investigated this hypothesis using the top 100 *R*_*gene*_ disease pairs (Supplementary Table 4). We divided the top 100 disease pairs into 10 groups with 10 pairs each, consecutively by the order. We merged *ΔR*_*gene*_ and *ΔDrug* of the 10 disease pairs in each group. We defined positive values (> 0) of *ΔR*_*gene*_ as surges and those of *ΔDrug* as active repositioning events. Then, by logistic regression analysis, we examined the association of *ΔR*_*gene*_ and *ΔDrug* in each group during the period. As a result, the surges of *R*_*gene*_ predicted active drug repositioning events occurring within 2 years, in all 10 groups with high odds ratio (3.89–13.34; Fig. 3C). Odds ratio was also high for those within a year (3.61–14.25) and within 3 years (2.01–19.14; Supplementary Fig. 5A–C).

Additionally, we examined the overall odds ratio of the top 100 disease pairs merged in one group. We found that, also in this case, the odds ratio for the predictability within 2 years was high (5.73), and it was also high for those within a year (6.52) and within 3 years (4.75; Supplementary Fig. 5D). Furthermore, we obtained similar results when we performed another analysis limiting data to Phase 3 and 4 clinical trials only (Supplementary Fig. 6).

These logistic regression analyses were performed as a sensitivity analysis to examine the predictability of drug repositioning events by *R*_*gene*_. The results confirmed that the surge of *R*_*gene*_ can predict active drug repositioning events, regardless of what disease pairs they are. Given this, the utility of *R*_*gene*_ in the prediction of drug repositioning events was validated.

## Discussion

In this study, we visualized the drug repositioning landscape in rare and intractable diseases. This landscape is rapidly expanding annually with a wide variety of inter-disease combinations. We invented a score, *R*_*gene*_, to investigate drug target gene-based proximity of a pair of diseases. *R*_*gene*_ represented the trend and potential of drug repositioning, and was useful in identifying the drug repositioning-active diseases. *R*_*gene*_ also potentiated the prediction of drug repositioning events. These findings illustrate the characteristics of drug repositioning trend and facilitate drug development in rare and intractable diseases.

Drug repositioning occurred frequently among rare and intractable diseases. In total, drug repositioning was identified between 4401 disease pairs in 1676 clinical trials. This large number of disease pairs is specific to the nature of rare and intractable diseases. The number of diseases is large, whereas the number of patients is small and the information available is limited. The diagnosis is often difficult, the pathogenic mechanism is unclear, and thus, available drugs are limited. The nature of rare and intractable diseases often interferes with novel drug developments, whereas drug repositioning may have compensated for the lack of drug developments. To cover broad ranges of diseases, drug repositioning has been attempted vigorously. On the other hand, the number of drug repositioning events were highly biased to a small number of disease pairs. The diseases frequently appeared in the disease pairs included such diseases as multiple sclerosis and Crohn’s disease. Most drug repositioning events were observed among these diseases which are large in number of patients. Less information of drug repositioning was available for most of rare and intractable diseases.

The degree of drug repositionability between a disease pair was further clarified using *R*_*gene*_. *R*_*gene*_ is a score for disease pairs sharing drug target genes which represent a common mechanism of drug action underling drug repositionability. In many cases, drugs were developed to target genes or gene products by inhibiting their disease-related activities as enzymes or receptors, to produce pharmacological effect^17,18^. Thus, if a disease pair shares more drug target genes, this disease pair is likely to share similar mechanisms of drug action. Although researchers in the world have vigorously applied drug repositioning in rare and intractable diseases, the lack of robust and sufficient information often hinders the identification of a suitable disease pair for drug repositioning. In rare and intractable diseases whose information is limited, investigation of drug target genes is crucial to access the mechanism of drug action. The repositionable disease pair is likely to share more target genes, denoting the closer drug target gene-based proximity of this pair. *R*_*gene*_ in a disease pair becomes high when the gene-based proximity becomes closer.

Besides its function to identify drug repositioning-active disease pairs, *R*_*gene*_ also elucidates the annual trends of drug repositioning among diseases. The number of repositioned drugs and *R*_*gene*_ increased during the observational period, indicating the accumulation of the number of drugs and drug target genes between a disease pair (MS–CD, Fig. 3A). In the trend analysis, we observed two waves of the annual changes of the number of repositioned drugs (*ΔDrug*) and *ΔR*_*gene*_ (Fig. 3B). *ΔR*_*gene*_ surges periodically, and these surges often preceded actual drug repositioning events (*ΔDrug*). The predictability of *R*_*gene*_ for the drug repositioning events was confirmed by logistic regression analyses throughout the top 100 drug repositioning-active disease pairs (Fig. 3C). *R*_*gene*_ predicted the potential of drug repositioning in the near future. Since *R*_*gene*_ is based on the sharedness of drug target genes between a disease pair and *ΔR*_*gene*_ surges before *ΔDrug*, a drug that targets the same gene was likely to be repositioned after the discovery of a new gene target of the drug.

*R*_*gene*_ provides a systematic approach to identify a target disease for repositioning, whereas *R*_*gene*_ has the potential for further improvement. Incorporation of information such as genetic variation and clinical phenotypes into *R*_*gene*_ may better represent the mechanism of drug action for targeting repositioning diseases. Modality and side effects of drugs are also important information that need to be incorporated for the information of drug repositioning. By accumulating testing data, cutoff values for *R*_*gene*_ in various cases can be determined to investigate the drug repositionability. Furthermore, since various drug combinations were tested in some clinical trials, *R*_*gene*_ will be applicable for the studies of pharmacodynamic drug interaction.

In many rare and intractable diseases, information on drug repositioning is still lacking. Development of the efficient methods to explore repositionable drugs in these diseases is still a challenge. Once the information of clinical trials and drug target genes are available, *R*_*gene*_ approach is efficient for this purpose. *R*_*gene*_ approach is also useful in other diseases, such as common diseases. Likewise, *R*_*gene*_ also provides the clue for new drug candidates not tested for rare and intractable diseases yet. Further drug repositioning is expected in the disease pairs which were identified by *R*_*gene*_ but had no repositioned drugs between them. *R*_*gene*_ approach is getting more effective these days, since target gene information is expanding. We expect that the invention of a score such as *R*_*gene*_ can broaden the drug repositioning landscape in rare and intractable diseases.

In conclusion, we visualized drug repositioning landscape in rare and intractable diseases. Drug target gene-based approach using *R*_*gene*_ provides both inter-disease and longitudinal perspectives as a whole landscape of drug repositioning, which is useful in drug development in rare and intractable diseases.

## Methods

### Data sources

Clinical trial data were obtained from four registries (JPRN^11^, ClinicalTrials.gov^12^, EU-CTR^13^, and ChiCTR^14^) through the website of the WHO International Clinical Trials Registry Platform^15^. Clinical trial data of the four registries were extracted from all clinical trial data in XML format and downloaded by searching WHO ICTRP with “Recruitment status: ALL" option. Drug data were downloaded from DrugBank^10^ (*drugbank_all_full_database.xml.zip*, version 5.1.4). Data on drug target genes were downloaded from KEGG^16^. In this study, data construction and analyses were conducted using our in-house Perl programs^19^ and other tools described later.

### Disease list

A list of rare and intractable diseases used in this study was initially created based on the 333 designated intractable diseases of the Ministry of Health, Labour and Welfare (MHLW) in Japan^20^. They included all fields of rare and intractable diseases covering 15 disease fields^9^. The list was manually constructed by translating Japanese disease names into English and assigning them synonyms and abbreviations. In addition, this list included second-level diseases (sub-categorized diseases), referenced in the MHLW documents. For example, lysosomal storage disease in the first level contains a subset of second-level diseases, such as Gaucher disease and Farber disease. In total, a list of 787 rare and intractable diseases consisting of a two-level categorized format (333 first-level diseases and 454 second-level diseases) was constructed (Supplementary Table 1).

### Data construction

Data construction was conducted as described in our previously reported method^21^ with some technical improvements for comprehensiveness. First, clinical trials with drug information were selected from the trials of the four registries. Two types of searches were conducted targeting the "Intervention" section of each trial data. (1) Searching for drug candidate descriptions by utilizing specific tags (e.g., "Drug:" for ClinicalTrials.gov) and (2) searching for drug names, synonyms, etc. based on DrugBank. Second, clinical trials for the rare and intractable diseases were selected by searching for the 787 disease names in "Public title", "Scientific title," and "Condition" sections of each trial data. These searches were conducted with detailed search options covering plural/singular forms, special characters (ä, ç, è, etc.), and word substitutions. For instance, for a disease name including “disease,” additional searches by substituting it with “disorder” or “syndrome” were also conducted. Some trials tested multiple diseases and/or multiple drugs, and some trials tested the same disease-drug combination.

Finally, from the clinical trials selected above, drugs were extracted and integrated to their target genes, referring to KEGG. First, drug data was connected between DrugBank and KEGG by referring links that appeared in each database and by searching drug names bi-directionally. Then, the extracted drugs were connected to their corresponding target genes using KEGG. If a drug had target gene information, the trial in which the drug was tested was selected to construct the data (Fig. 1A).

### Drug repositioning detection

Detection of drug repositioning was conducted among 333 first-level diseases. Drugs were assigned to each disease based on clinical trial data. Then, for each disease pair (A, B), the numbers of shared drugs (A and B), and unshared drugs (A only or B only) were counted. Each disease pair sharing at least one drug target gene was determined as a repositionable disease pair to be analyzed. Based on the “Date of first enrolment” section of clinical trial data, we constructed annual data of a set of clinical trials enrolled in and before each year from 2000 to 2019 (~ Nov. 2019). For example, the annual data of 2008 contain the clinical trials enrolled both in 2008 and before 2008. For each annual data, the numbers of shared and unshared drugs were counted per disease pair. Then, for each disease pair, differences in the number of drugs between every two consecutive years were counted.

Drug repositioning was defined as follows: in a disease pair (A, B), if an unshared drug in disease A becomes newly shared in the next year, then this is a drug repositioning event where the drug was repositioned from disease A (donor) to disease B (acceptor; Fig. 1B). For each disease pair (A, B), annual changes in the number of repositioned drugs were defined as *ΔDrug*. The number of repositioned drugs for both “A to B” and “B to A” repositioning was counted. *ΔDrug* standardization was conducted as follows: (*ΔDrug_*_*i*_–*av*) / *SD,* where *ΔDrug_*_*i*_, *av*, and *SD* are *ΔDrug* for each year, an average of *ΔDrug*, and standard deviation, respectively, for each disease pair with *SD* > 0.

### ***R***_***gene***_ calculation

The numbers of drug target genes were counted as well for each disease pair by converting drugs to their target genes (Fig. 2A). Based on the numbers of shared and unshared genes, *R*_*gene*_ was calculated for each disease pair using the formula shown in Fig. 2B. The disease pairs for which *R*_*gene*_ > 0 were expanded to a disease network. A circular network shown in Fig. 2D was depicted by Cytoscape v3.8.2 (Degree Sorted Circle Layout)^22^. *ΔR*_*gene*_ and its standardization was conducted in the same way as *ΔDrug*.

### Odds ratio for ***ΔR***_***gene***_ and ***ΔDrug***

Logistic regression analyses were conducted to calculate the odds ratio and confidence intervals for the association between *ΔR*_*gene*_ and *ΔDrug*. They were conducted for disease pairs in which *ΔR*_*gene*_ was calculated in two or more years, and when at least one drug repositioning event occurred during the period. Before calculation, *ΔR*_*gene*_ and *ΔDrug* were converted to 1 (if > 0) or 0 (if ≤ 0), and additionally two sets of *ΔDrug* were prepared: *ΔDrug*_+1_ = 1 if *ΔDrug* = 1 in the same year or next year, and *ΔDrug*_+2_ = 1 if *ΔDrug* = 1 in the same year or within the next two years. Odds ratio was calculated for (1) *ΔR*_*gene*_ and *ΔDrug*, (2) *ΔR*_*gene*_ and *ΔDrug*_+1_, and (3) *ΔR*_*gene*_ and *ΔDrug*_+2_. Odds ratios were calculated for each set of 10 disease pairs in the top 100 *R*_*gene*_ disease pairs, from the top to the last. The analyses were conducted by *logistic.display* in the *epiDisplay* library of R version 4.0.3^23^.

## Supplementary Information

Supplementary Information.

## Data Availability

The datasets used and/or analyzed during the current study are available from the supplementary information files and the database (DDrare: Database of Drug Development for Rare Diseases: https://ddrare.nibiohn.go.jp/index_e.html).
