# Supplementary material for: Drug target gene-based analyses of drug repositionability in rare and intractable diseases
Source: Sci Rep. 2021 Jun 11;11:12338. doi: 10.1038/s41598-021-91428-4 (PMC8196006; doi:10.1038/s41598-021-91428-4)
Supplement: Supplementary file 1 — Supplementary Information. [file 41598_2021_91428_MOESM1_ESM.pdf]

Drug Target Gene-based Analyses of Drug Repositionability in Rare and Intractable Diseases

Ryuichi Sakate[1,2*], Tomonori Kimura[1,2,3,4*]

[1] Laboratory of Rare Disease Resource library, Center for Rare Disease Research, National Institutes of Biomedical Innovation, Health and Nutrition (NIBIOHN)

[2] Platform of Therapeutics for Rare Disease, Center for Rare Disease Research, National Institutes of Biomedical Innovation, Health and Nutrition (NIBIOHN)

[3] Reverse Translational Research Project, Center for Rare Disease Research, National Institutes of Biomedical Innovation, Health and Nutrition (NIBIOHN)

[4] KAGAMI Project, National Institutes of Biomedical Innovation, Health and Nutrition (NIBIOHN)

*To whom correspondence should be addressed to: TK (t-kimura@nibiohn.go.jp); RS (rsakate@nibiohn.go.jp)

Supplementary Figure 1-6
and
Supplementary Table 1-5

Supplementary Figure 1

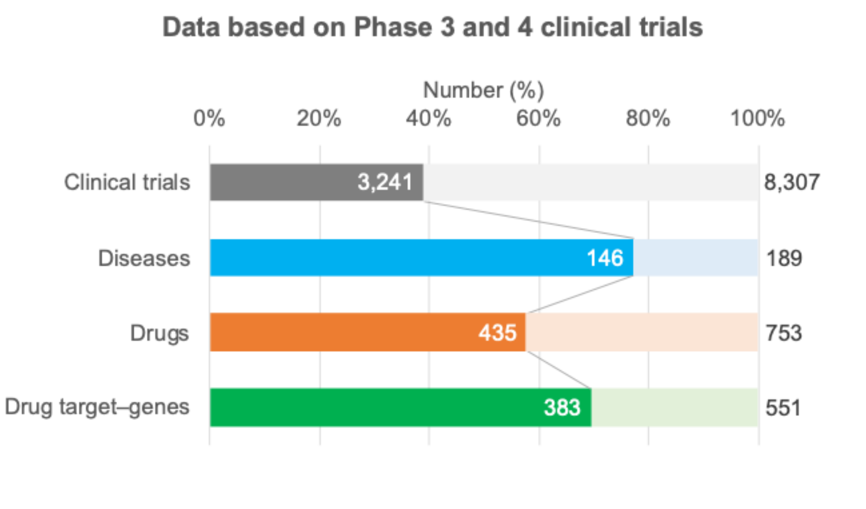

Supplementary Figure 1. Data of Phase 3 and 4 clinical trials (3,241 out of 8,307 trials): Distribution of the number of trials, diseases, drugs, and drug target genes.

Supplementary Figure 2

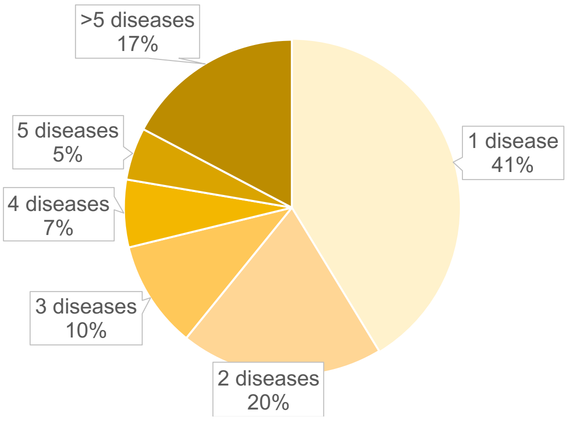

Supplementary Figure 2. Out of 753 drugs, 442 (59%) were tested for two or more diseases in clinical trials.

Supplementary Figure 3

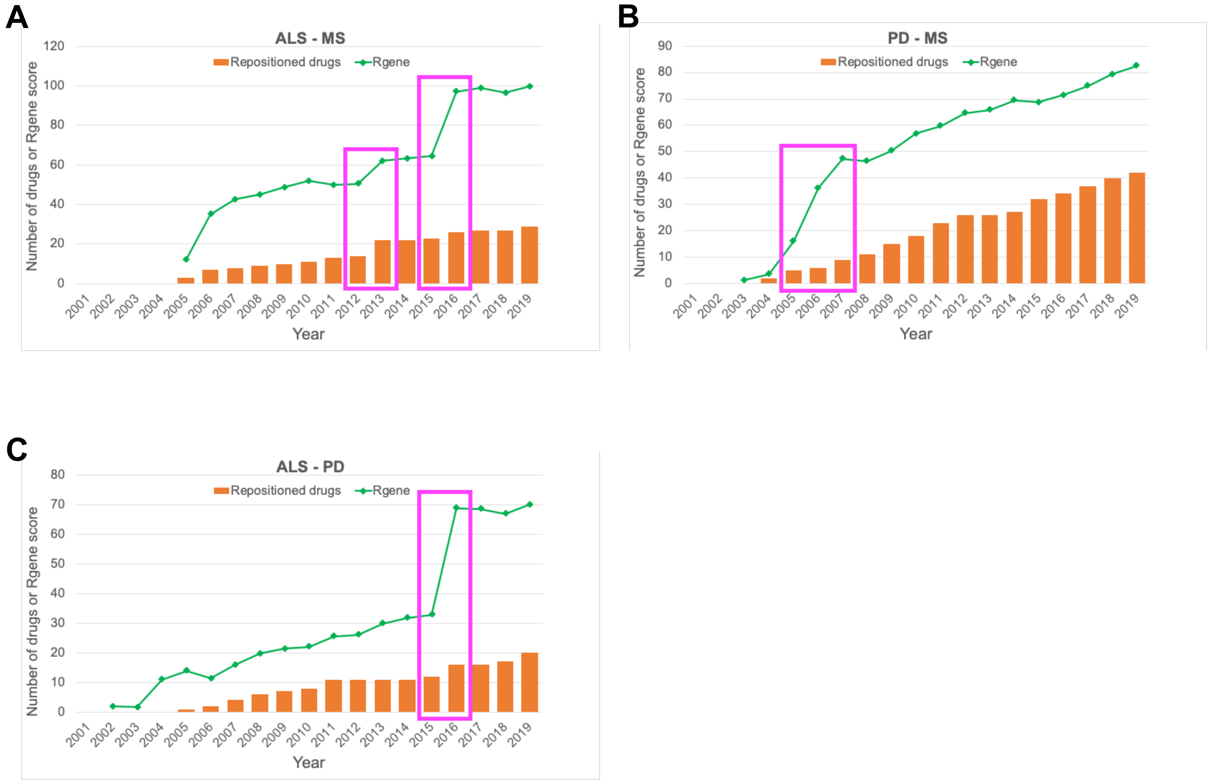

Supplementary Figure 3. Annual changes in repositioned drugs and R_{gene} for three disease pairs. **(A)** Amyotrophic lateral sclerosis (ALS) – Multiple sclerosis (MS), **(B)** Parkinson's disease (PD) – Multiple sclerosis (MS), **(C)** Amyotrophic lateral sclerosis (ALS) – Parkinson's disease (PD).

Supplementary Figure 4

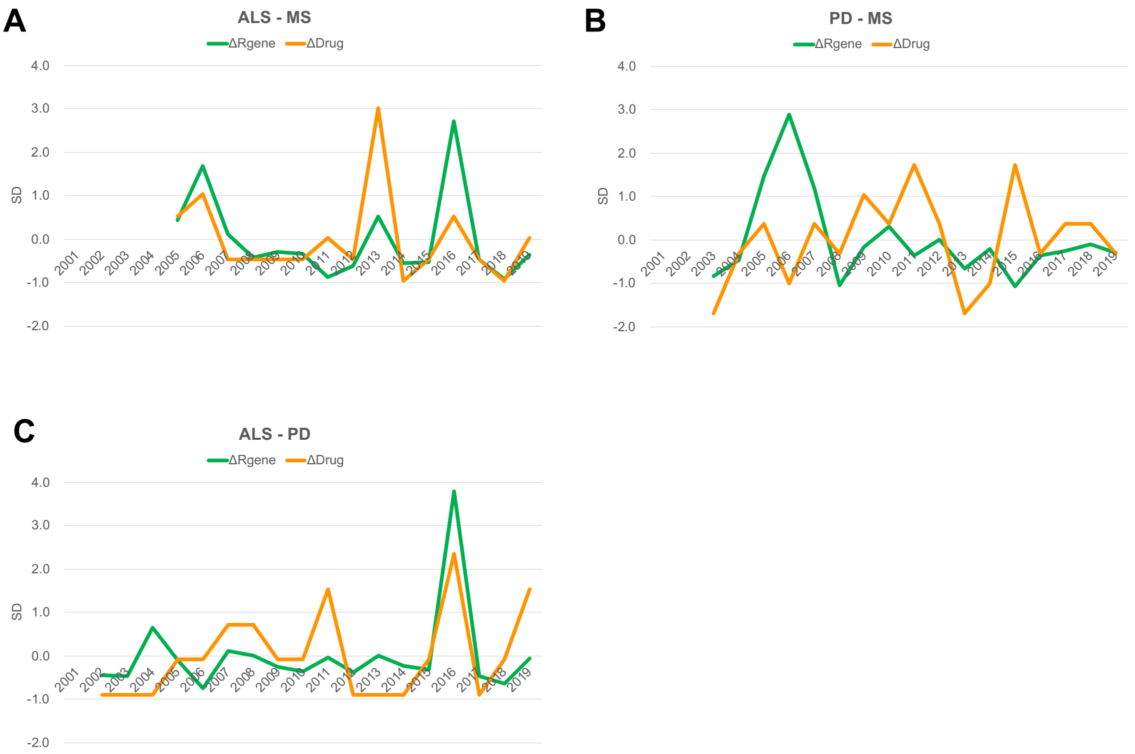

Supplementary Figure 4. Annual changes in repositioned drugs and R_{gene} for three disease pairs. **(A)** Amyotrophic lateral sclerosis (ALS) – Multiple sclerosis (MS), **(B)** Parkinson's disease (PD) – Multiple sclerosis (MS), **(C)** Amyotrophic lateral sclerosis (ALS) – Parkinson's disease (PD).

Supplementary Figure 5

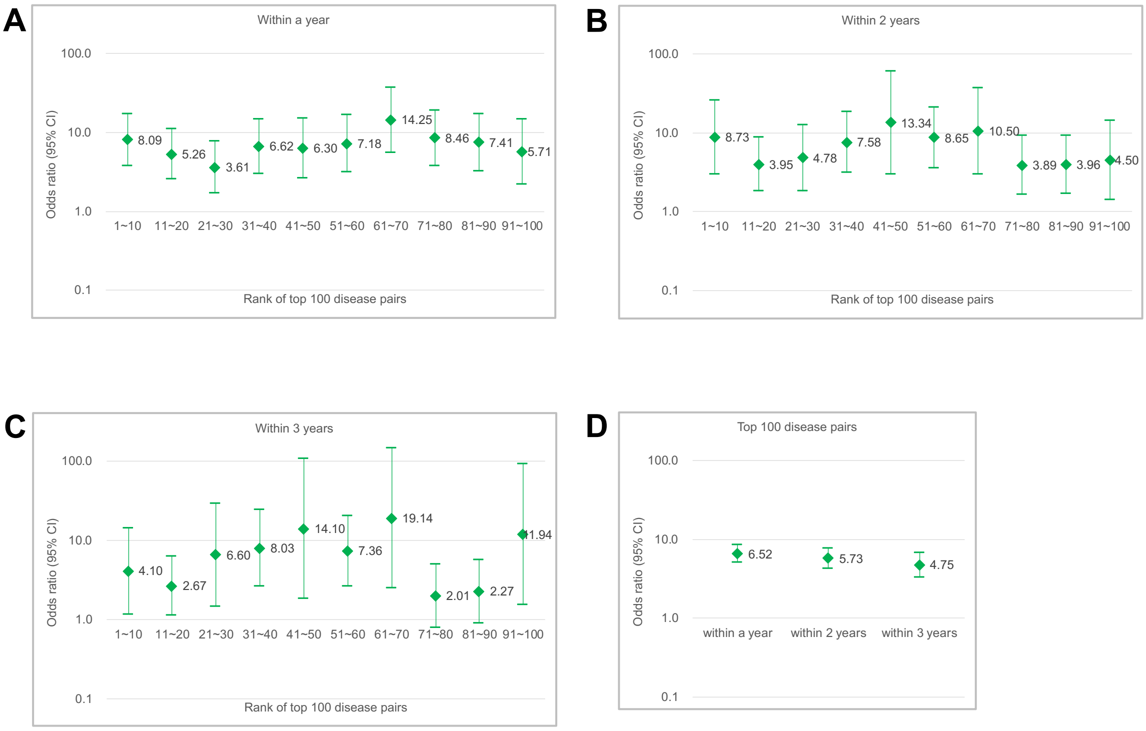

Supplementary Figure 5. Odds ratios of ΔR_{gene} and $\Delta Drug$ as an explanatory variable and an objective variable, respectively, were analyzed by logistic regression analyses for the top 100 R_{gene} disease pairs. **(A-C)** The 100 disease pairs were divided into 10 groups consecutively by the order. For the surge of R_{gene}, three patterns were analyzed whether active repositioning events occur **(A)** within a year (the same year), **(B)** within 2 years, and **(C)** within 3 years. **(D)** The 100 disease pairs were analyzed as one group. Three patterns were also analyzed. Note: In fact, 93 disease pairs were used because 7 pairs lacked drug repositioning events (Supplemental Table 4).

Supplementary Figure 6

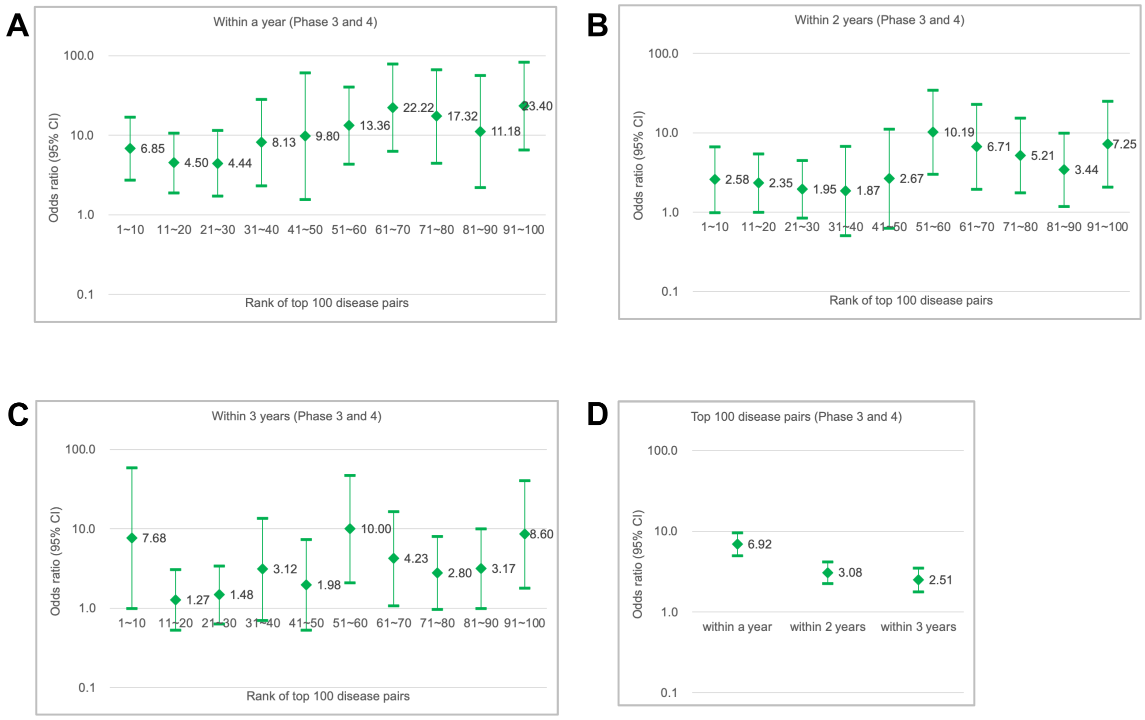

Supplementary Figure 6. Odds ratios of *⊿*R_{gene} and *⊿Drug* as an explanatory variable and an objective variable, respectively, based on the data of Phase 3 and 4 clinical trials only. The same analyses as shown in Supplementary Figure 5 were conducted. Note: 70 disease pairs were used because 30 pairs lacked drug repositioning events or Phase 3 or 4 clinical trials (Supplemental Table 4).

Supplementary Table 1. The rare and intractable diseases based on the 333 resignated intractable diseases in Japan, assigned with subcategorized diseases, synonyms and abbreviations.

15 disease fields: A. Neuromuscular diseases, B. Metabolic diseases, C. Cutaneous connective tissue diseases, D. Immune diseases, E. Cardiovascular diseases, F. Blood diseases, G. Renal and urological diseases, H. Bone and joint diseases, I. Endocrine diseases, J. Respiratory diseases, K. Eye diseases, L. Hearing and balance diseases, M. Digestive diseases, N. Chromosomal or genetic disorders, O. Otolaryngologic diseases

ID	Disease field	Disease name
1	A	Spinal and bulbar muscular atrophy;Spinobulbar muscular atrophy;Kennedy disease;
2	A	Amyotrophic lateral sclerosis;
3	A	Spinal muscular atrophy;Myelopathic muscular atrophy;
3-1	A	Spinal muscular atrophy type I;SMA I;Werdnig-Hoffman disease;
3-2	A	Spinal muscular atrophy type II;SMA II;Dubowitz disease;
3-3	A	Spinal muscular atrophy type III;SMA III;Kugelberg-Welander disease;
3-4	A	Spinal muscular atrophy type IV;SMA IV;
4	A	Primary lateral sclerosis;
5	A	Progressive supranuclear palsy;
6	A	Parkinson disease;
7	A	Corticobasal degeneration;Corticobasal syndrome;
8	A	Huntington disease;Huntington chorea;
9	A	Neuroacanthocytosis;
9-1	A	Choreoacanthocytosis;Chorea-acanthocytosis;Levine-Critchley syndrome;
9-2	A	McLeod syndrome;
10	A	Charcot-Marie-Tooth disease;
11	A	Myasthenia gravis;
12	A	Congenital myasthenic syndrome;
12-1	A	End-plate acetylcholine receptor deficiency;
12-2	A	Slow-channel congenital myasthenic syndrome;
12-3	A	Fast-channel congenital myasthenic syndrome;
12-4	A	Sodium channel myasthenia;
12-5	A	End-plate acetylcholine esterase deficiency;
12-6	A	Congenital myasthenic syndrome with episodic apnoea;
13	A	Multiple sclerosis;Neuromyelitis optica;
13-1	A	Multiple sclerosis;
13-2	A	Neuromyelitis optica;
13-3	A	Devic disease;
13-4	A	Balo concentric sclerosis;Baló concentric sclerosis;
14	A	Chronic inflammatory demyelinating polyneuropathy;Chronic inflammatory demyelinating poly (radiculo) neuropathy;CIDP;Multifocal motor neuropathy;
14-1	A	Chronic inflammatory demyelinating polyneuropathy;Chronic inflammatory demyelinating poly (radiculo) neuropathy;CIDP;
14-2	A	Multifocal motor neuropathy;
15	A	Inclusion body myositis;
16	A	Crow-Fukase syndrome;POEMS syndrome;
16-1	A	Polyneuropathy, organomegaly, endocrinopathy, m-protein, and skin changes syndrome;POEMS syndrome;
16-2	A	Takatsuki disease;
16-3	A	PEP syndrome;Polyneuropathy, endocrinopathy, plasma cell dyscrasia syndrome;
17	A	Multiple system atrophy;
17-1	A	Olivopontocerebellar atrophy;
17-2	A	Striatonigral degeneration;
17-3	A	Shy-Drager syndrome;
18	A	Spinocerebellar degeneration;
19	B	Lysosomal storage disease;Lysosomal disease;
19-1	B	Gaucher disease;
19-2	B	Niemann-Pick disease;
19-3	B	Niemann-Pick disease type C;Niemann-Pick disease, type C;Niemann-Pick type C;
19-4	B	GM1-gangliosidosis;GM1-gangliosidoses;
19-5	B	GM2-gangliosidosis;GM2-gangliosidoses;Tay-Sachs disease;Sandhoff disease;
19-6	B	Krabbe disease;
19-7	B	Metachromatic leukodystrophy;
19-8	B	Multiple-sulfatase deficiency;
19-9	B	Farber disease;
19-10	B	Mucopolysaccharidosis type I;Mucopolysaccharidosis I;MPS I;Hurler syndrome;Hurler-Scheie syndrome;Scheie syndrome;
19-11	B	Mucopolysaccharidosis type II;Mucopolysaccharidosis II;MPS II;Hunter syndrome;
19-12	B	Mucopolysaccharidosis type III;Mucopolysaccharidosis III;MPS III;Sanfilippo syndrome;
19-13	B	Mucopolysaccharidosis type IV;Mucopolysaccharidosis IV;MPS IV;MPS IVA;Morquio syndrome;Morquio A syndrome;
19-14	B	Mucopolysaccharidosis type VI;Mucopolysaccharidosis VI;MPS VI;Maroteaux-Lamy syndrome;
19-15	B	Mucopolysaccharidosis type VII;Mucopolysaccharidosis VII;MPS VII;Sly syndrome;
19-16	B	Mucopolysaccharidosis type IX;Mucopolysaccharidosis IX;MPS IX;Hyaluronidase deficiency;
19-17	B	Sialidosis;
19-18	B	Galactosialidosis;

19-19	B	Mucolipidosis II;Mucolipidosis type II;I-cell disease;Mucolipidosis III;Mucolipidosis type III;
19-20	B	Alpha-Mannosidosis;Alpha-Mannosidase Deficiency;
19-21	B	Beta-Mannosidosis;Beta-Mannosidase Deficiency;
19-22	B	Fucosidosis;
19-23	B	Aspartylglucosaminuria;
19-24	B	Schindler disease;Kanzaki disease;
19-25	B	Pompe disease;
19-26	B	Acid lipase deficiency;Wolman disease;Cholesterol ester storage disease;
19-27	B	Danon disease;
19-28	B	Free sialic acid storage disease;Salla disease;
19-29	B	Ceroid lipofuscinosis;
19-30	B	Fabry disease;
19-31	B	Cystinosis;
20	B	Adrenoleukodystrophy;
20-1	B	Adrenomyeloneuropathy;
21	B	Mitochondrial disease;
22	A	Moyamoya disease;Occlusive disease in circle of Willis;
23	A	Prion disease;
23-1	A	Creutzfeldt-Jakob disease;
23-2	A	Gerstmann-Straussler-Scheinker syndrome;
23-3	A	Fatal familial insomnia;
23-4	A	Kuru disease;
24	A	Subacute sclerosing panencephalitis;
25	A	Progressive multifocal leukoencephalopathy;Leukoencephalopathy, progressive multifocal;
26	A	HTLV-1-associated myelopathy;Tropical spastic paraparesis;HTLV-1;HTLV-I-associated myelopathy;
27	A	Idiopathic basal ganglia calcification;Idiopathic basal ganglia calcification disease;
27-1	A	Fahr disease;
27-2	A	Familial idiopathic basal ganglia calcification;FIBGC;
27-3	A	Primary familial brain calcification;PFBC;
28	B	Systemic amyloidosis;AL amyloidosis;
28-1	B	Immunoglobulin light chain amyloidosis;Amyloid light-chain amyloidosis;AL amyloidosis;
28-2	B	Familial amyloid polyneuropathy;
28-3	B	Senile systemic amylodosis;
29	A	Ullrich disease;Ullrich congenital muscular dystrophy;Collagen VI-related myopathy;
30	A	Distal myopathy;Distal muscular dystrophy;
30-1	A	Miyoshi myopathy;
30-2	A	Distal myopathy with rimmed vacuoles;
30-3	A	Oculopharyngodistal myopathy;
31	A	Bethlem myopathy;Beth Rem myopathy;
32	A	Autophagic vacuolar myopathy;
32-1	A	Danon disease;
32-2	A	X-linked myopathy with excessive autophagy;XMEA;
33	A	Schwartz-Jampel syndrome;Schwarz-Yanperu syndrome;Myotonic chondrodystrophy;Cartilage dystrophic myotonia;
33-1	A	Stuve-Wiedemann syndrome;Stüve-Wiedemann syndrome;
34	C	Neurofibromatosis;
34-1	C	von Recklinghausen disease;
35	C	Pemphigus;
36	C	Epidermolysis bullosa;
36-1	C	Kindler syndrome;
37	C	Generalised pustular psoriasis;Pustular psoriasis;
37-1	C	Acute generalised pustular psoriasis, von Zumbusch type;
37-2	C	Herpetic impetigo;
38	C	Stevens-Johnson syndrome;Mucocutaneous ocular syndrome;
39	C	Toxic epidermal necrolysis;Toxic epidermal necrosis;
40	D	Takayasu arteritis;Aortitis syndrome;Pulseless disease;
41	D	Giant cell arteritis;Temporal arteritis;
42	D	Polyarteritis nodosa;
43	D	Microscopic polyangiitis;
44	D	Wegener granulomatosis;Multiple vasculitis granulomatous disease;Granulomatosis with polyangiitis;
45	D	Eosinophilic granulomatosis with Polyangiitis;EGPA;Eosinophilic multiple vasculitis granulomatous disease;Allergic granulomatous angiitis;Churg-Strauss syndrome;
46	D	Malignant rheumatoid arthritis;Rheumatoid arthritis;Rheumatoid arthritis with vasculitis;
47	D	Buerger disease;Thromboangiitis obliterans;
48	D	Primary antiphospholipid syndrome;Primary antiphospholipid antibody syndrome;
49	D	Systemic lupus erythematosus;
50	D	Dermatomyositis;Polymyositis;
50-1	D	Dermatomyositis;
50-2	D	Polymyositis;

51	C	Systemic scleroderma;Systemic sclerosis;
52	CD	Mixed connective tissue disease;
53	D	Sjogren syndrome;Sjögren syndrome;
54	D	Adult still disease;Adult-onset Stills disease;
55	D	Relapsing polychondritis;
56	D	Behcet disease;Behçet disease;
57	E	Idiopathic dilated cardiomyopathy;
58	E	Hypertrophic cardiomyopathy;
59	E	Restricted cardiomyopathy;Restrictive cardiomyopathy;Constrictive cardiomyopathy;
60	F	Aplastic anemia;
61	F	Autoimmune hemolytic anemia;AIHA;
61-1	F	Cold agglutinin disease;
61-2	F	Paroxysmal cold hemoglobinuria;
62	F	Paroxysmal nocturnal hemoglobinuria;
63	F	Idiopathic thrombocytopenic purpura;Primary immune thrombocytopenia;
64	F	Thrombotic thrombocytopenic purpura;
64-1	F	Upshaw-Schulman syndrome;
65	F	Primary immunodeficiency;
65-1	F	X-linked severe combined immunodeficiency;X-SCID;
65-2	F	Reticular dysgenesis;
65-3	F	Adenosine deaminase deficiency;
65-4	F	Omenn syndrome;
65-5	F	Purine nucleoside phosphorylase deficiency;
65-6	F	CD8 deficiency;
65-7	F	ZAP-70 deficiency;
65-8	F	MHC class I deficiency;
65-9	F	MHC class II deficiency;
65-10	F	Combined immunodeficiency;
65-11	F	Wiskott-Aldrich syndrome;
65-12	F	Telangiectasia ataxia;
65-13	F	Nijmegen breakage syndrome;
65-14	F	Bloom syndrome;
65-15	F	Immunodeficiency, centromere region instability, facial anomalies syndrome;ICF syndrome;
65-16	F	PMS2 deficiency;
65-17	F	Radiosensitivity, immunodeficiency, dysmorphic features, and learning difficulties syndrome;RIDDLE syndrome;
65-18	F	Schimke syndrome;
65-19	F	Netherton syndrome;
65-20	F	Thymic hypoplasia;DiGeorge syndrome;22q11.2 deletion syndrome;
65-21	F	Hyper-IgE syndrome;
65-22	F	Hepatic venoocclusive immunodeficiency;Immunodeficiency with central hepatic vein atresia;
65-23	F	Dyskeratosis congenita;
65-24	F	X-linked agammaglobulinaemia;
65-25	F	Common variable immunodeficiency;
65-26	F	Hyper-IgM syndrome;
65-27	F	Isolated IgG subclass deficiency;
65-28	F	Selective IgA deficiency;
65-29	F	Specific antibody production deficiency;
65-30	F	Infant transient hypogammaglobulinemia;
65-31	F	Chédiak-Higashi syndrome;Chediak-Higashi syndrome;
65-32	F	X-linked lymphoproliferative syndrome;
65-33	F	SAP deficiency;SH2D1A/SLAM-associated protein deficiency;
65-34	F	XIAP deficiency;X-linked inhibitor of apoptosis deficiency;
65-35	F	Autoimmune lymphoproliferative syndrome;ALPS;
65-36	F	Familial hemophagocytic syndrome;Perforin deficiency;Munc13-4 deficiency;Syntaxin 11 deficiency;Munc18-2 deficiency;
65-37	F	Autoimmune polyendocrinopathy-candidiasis-ectodermal dystrophy;APECED;
65-38	F	Immune dysregulation, polyendocrinopathy, enteropathy, X-linked syndrome;IPEX syndrome;
65-39	F	CD25 deficiency;
65-40	F	ITCH deficiency;
65-41	F	Primary phagocytic dysfunction;
65-42	F	Severe congenital neutropenia;
65-43	F	Cyclic neutropenia;
65-44	F	Hermanskyi-Pudlak syndrome type 2;Hermanskyi-Pudlak syndrome 2;
65-45	F	Griscelli syndrome type 2;Griscelli syndrome 2;
65-46	F	p14 deficiency;
65-47	F	Warts, hypogammaglobulinemia, infections, myelokathexis syndrome;WHIM syndrome;
65-48	F	Glycogen storage disease type Ib;
65-49	F	Leukocyte adhesion deficiency;
65-50	F	Shwachman-Diamond syndrome;

65-51	F	Chronic granulomatous disease;
65-52	F	Myeloperoxidase deficiency;
65-53	F	Mendelian susceptibility to mycobacterial disease;MSMD;
65-54	F	Anhidrotic ectodermal dysplasia with immunodeficiency;EDA-ID;
65-55	F	Interleukin-1 receptor-associated kinase-4 deficiency;IRAK4 deficiency;
65-56	F	IMyD88 deficiency;
65-57	F	Chronic mucocutaneous candidiasis;
65-58	F	Epidermodysplasia verruciformis;
65-59	F	Herpes simplex encephalitis;
65-60	F	Caspase recruitment domain family member 9 deficiency;CARD9 deficiency;
65-61	F	Trypanosomiasis;
65-62	F	Congenital complement deficiency;
65-63	F	C1q deficiency;
65-64	F	CC1r deficiency;
65-65	F	CC1s deficiency;
65-66	F	CC2 deficiency;
65-67	F	CC3 deficiency;
65-68	F	CC4 deficiency;
65-69	F	CC5 deficiency;
65-70	F	CC6 deficiency;
65-71	F	CC7 deficiency;
65-72	F	CC8 deficiency;
65-73	F	CC9 deficiency;
65-74	F	Factor D deficiency;
65-75	F	Properdin deficiency;
65-76	F	Factor I deficiency;
65-77	F	Factor H deficiency;
65-78	F	MASP1 deficiency;
65-79	F	3MC syndrome;
65-80	F	Mannose-binding protein-associated serine protease 2 deficiency;MASP2 deficiency;
65-81	F	Immunodeficiency associated with FCN3 mutation;FCN3;
65-82	F	Hereditary angioedema type 1;Hereditary angioedema type I;C1 inhibitor deficiency type 1;C1 inhibitor deficiency type I;
65-83	F	Hereditary angioedema type 2;Hereditary angioedema type II;C1 inhibitor deficiency type 2;C1 inhibitor deficiency type II;
65-84	F	Hereditary angioedema type 3;Hereditary angioedema type III;C1 inhibitor deficiency type 3;C1 inhibitor deficiency type III;
66	G	IgA nephropathy;IgA nephritis;Berger disease;IgA-IgG nephropathy;
67	G	Polycystic kidney;
68	H	Ossification of the ligamentum flavum;Ossification of ligamentum flavum;
69	H	Ossification of posterior longitudinal ligament;
70	H	Spinal stenosis;Extensive spinal canal stenosis;
71	H	Idiopathic osteonecrosis of the femoral head;Idiopathic femoral head necrosis;
72	I	Pituitary ADH secretion disorder;Inappropriate antidiuretic hormone secretion;Syndrome of inappropriate secretion of antidiuretic hormone;Inappropriate ADH syndrome;Syndrome of inappropriate ADH;
72-1	I	Central diabetes insipidus;
72-2	I	Syndrome of inappropriate secretion of ADH;SIADH;
73	I	TSH-secreting pituitary adenoma;Pituitary TSH secretion hyperthyroidism;
74	I	Prolactin secreting pituitary adenoma;Pituitary PRL secretion hyperthyroidism;Prolactinoma;Prolactin secreting adenoma;
75	I	Cushing disease;Cushing;
76	I	Pituitary gonadotropin secretion hyperthyroidism;Gonadotropin secreting pituitary adenoma;
76-1	I	Central precocious puberty;
76-2	I	Gonadotropin producing pituitary adenoma;
77	I	Growth hormone secreting pituitary adenoma;Pituitary growth hormone secretion hyperthyroidism;
78	I	Hypopituitarism;Anterior pituitary hypothyroidism;
78-1	I	Syndrome of abnormal secretion of gonadotropin;Hyposecretion of gonadotropins;
78-2	I	Adrenocorticotropic hormone deficiency;ACTH deficiency;
78-3	I	Thyroid-stimulating hormone deficiency;TSH deficiency;
78-4	I	Growth hormone deficiency;GH deficiency;
78-5	I	Prolactin deficiency;PRL deficiency;
79	B	Homozygous familial hypercholesterolemia;
80	I	Resistance to thyroid hormone;Syndrome of resistance to thyroid hormone;Thyroid hormone insensitivity syndrome;Refetoff syndrome;
81	I	Congenital adrenal hyperplasia;Congenital adrenal enzyme deficiency;Congenial adrenal cortex enzyme deficiency;
81-1	I	Congenital Lipoid Adrenal Hyperplasia;
81-2	I	3β-Hydroxysteroid Dehydrogenase Deficiency;
81-3	I	21-Hydroxylase deficiency;
81-4	I	11β-Hydroxylase deficiency;
81-5	I	17α-Hydroxylase deficiency;
81-6	I	Aldosterone synthase deficiency;
81-7	I	P450 oxidoreductase deficiency;
82	I	Congenital adrenal hypoplasia;

82-1	I	X-linked congenital adrenal hypoplasia;DAX-1 deficiency;
82-2	I	Congenital adrenal hypoplasia, autosomal recessive form;Steroidogenic factor-1 deficiency;SF-1 deficiency;SF-1/Ad4BP deficiency;
82-3	I	IMAGe syndrome;
83	I	Addison disease;Primary chronic adrenocortical insufficiency;
83-1	I	Autoimmune polyglandular syndrome;
83-2	I	Hypoparathyroidism-Addison-Monilia syndrome;HAM syndrome;
83-3	I	Schmidt syndrome;
84	J	Sarcoidosis;
85	J	Idiopathic interstitial pneumonia;
85-1	J	Idiopathic pulmonary fibrosis;
85-2	J	Usual interstitial pneumonia;
85-3	J	Non-specific interstitial pneumonia;NSIP;
85-4	J	Cryptogenic organizing pneumonia;
85-5	J	Organizing pneumonia;
85-6	J	Desquamative interstitial pneumonia;
85-7	J	Respiratory bronchiolitis - associated interstitial lung disease;RB-ILD;
85-8	J	Lymphocytic interstitial pneumonia;
85-9	J	Acute interstitial pneumonia;
85-10	J	Diffuse alveolar damage;
86	J	Pulmonary arterial hypertension;
86-1	J	Eisenmenger Syndrome;
87	J	Pulmonary veno-occlusive disease;Pulmonary capillary hemangiomatosis;
87-1	J	Pulmonary veno-occlusive disease;PVOD;
87-2	J	Pulmonary capillary hemangiomatosis;
88	J	Chronic thromboembolic pulmonary hypertension;CTEPH;Idiopathic chronic pulmonary thromboembolism;
89	J	Lymphangioleiomyomatosis;
90	K	Retinitis pigmentosa;
90-1	K	Rod dystrophy;
90-2	K	Cone-rod dystrophy;Rod-Cone Dystrophy;
91	M	Budd-Chiari syndrome;
92	M	Idiopathic portal hypertension;Banti syndrome;
93	M	Primary biliary cholangitis;Primary biliary cirrhosis;
94	M	Primary sclerosing cholangitis;
95	M	Autoimmune hepatitis;
96	M	Crohn disease;Terminal ileitis;
97	M	Ulcerative colitis;
98	M	Eosinophilic gastrointestinal disease;Eosinophilic gastroenteritis;Eosinophilic esophagitis;Eosinophilic colitis;Eosinophilic gastro-intestinal disorder;EGID;
98-1	M	Neonatal food-protein induced enterocolitis;Neonatal Food-protein induced enterocolitis syndrome;N-FPIES;
98-2	M	Eosinophilic esophagitis;
98-3	M	Eosinophilic gastroenteritis;
99	M	Chronic intestinal pseudo-obstruction;Chronic idiopathic pseudo-bowel obstruction;
100	M	Megacystis microcolon intestinal hypoperistalsis syndrome;Huge bladder short and small colon intestinal peristalsis deficiency;
101	M	Congenital isolated hypoganglionosis;Intestinal ganglion cells insignificant disease;
102	N	Rubinstein-Taybi syndrome;RSTS;
103	N	Cardio-facio-cutaneous syndrome;CFC syndrome;
104	N	Costello syndrome;
105	N	CHARGE syndrome;
106	D	Cryopyrin-associated periodic syndrome;Cryopyrin associated periodic fever syndrome;
106-1	D	Familial cold autoinflammatory syndrome;FCAS;
106-2	D	Mucke-Wells syndrome;
106-3	D	Chronic infantile neurologic cutaneous, and articular syndrome;CINCA syndrome;
106-4	D	Neonatal onset multisystem inflammatory disease;NOMID;
107	D	Systemic juvenile idiopathic arthritis;Systemic-onset juvenile idiopathic arthritis;
108	D	TNF receptor-associated periodic syndrome;
109	G	Atypical hemolytic uremic syndrome;
110	D	Blau syndrome;Early-onset sarcoidosis;Systemic granulomatous diseases;
111	A	Congenital myopathy;
111-1	A	Nemaline myopathy;
111-2	A	Central core disease;
111-3	A	Minicore myopathy;
111-4	A	Multi-minicore myopathy;
111-5	A	Myotubular myopathy;
111-6	A	Centronuclear myopathy;
111-7	A	Congenital fiber-type disproportion myopathy;
112	A	Marinesco-Sjogren syndrome;Hereditary cerebellar ataxia-childhood cataracts;
113	A	Muscular dystrophy;
113-1	A	Dystrophinopathies;
113-2	A	Duchenne muscular dystrophy;

113-3	A	Becker muscular dystrophy;
113-4	A	Limb-girdle muscular dystrophy;
113-5	A	Myotilinopathy;
113-6	A	Laminopathy;
113-7	A	Caveolinopathy;Limb gridle muscular dystrophy 1C;LGMD1C;
113-8	A	Desminopathy;
113-9	A	Sarcoglycanopathy;
113-10	A	α-dystroglycanopathy;
113-11	A	Congenital muscular dystrophy;
113-12	A	Facioscapulohumeral muscular dystrophy;
113-13	A	Emery-Dreifuss muscular dystrophy;
113-14	A	Oculopharyngeal muscular dystrophy;
113-15	A	Fukuyama-type congenital muscular dystrophy;FCMD;
113-16	A	Walker-Warburg syndrome;
113-17	A	Muscle-eye-brain disease;
113-18	A	Myotonic dystrophy;
113-19	A	Integrin α7 deficient CMD;CIntegrin α7 deficient ongenital muscular dystrophy;
113-20	A	Merosin-deficient congenital muscular dystrophy;
113-21	A	Ullrich congenital muscular dystrophy;
113-22	A	Rigid spine syndrome;
113-23	A	Dynamin 2 deficient congenital muscular dystrophy;
113-24	A	Telesonin-deficient congenital muscular dystrophy;
113-25	A	Congenital muscular dystrophy with mitochondrial structural abnormalities;
114	A	Non-dystrophic myotonia syndrome;Non-dystrophic Myotonia;
114-1	A	Myotonia congenita;
114-2	A	Paramyotonia congenita;
114-3	A	Thomsen disease;Autosomal-dominant myotonia congenita;
114-4	A	Becker disease;Autosomal-recessive myotonia congenita;
114-5	A	Sodium channel myotonia;
115	A	Hereditary periodic paralysis;
115-1	A	Hereditary Hypokalemic Periodic Paralysis;
115-2	A	Hereditary Hyperkalemic Periodic Paralysis;
115-3	A	Andersen-Tawil syndrome;
116	A	Atopic myelitis;Idiopathic eosinophilic myelitis;
117	A	Syringomyelia;
118	A	Myelomeningocele;
118-1	A	Myeloschisis;
118-2	A	Myelocele;
118-3	A	Myelocystocele;Syringomyelocele;
119	A	Isaacs syndrome;
119-1	A	Morvan syndrome;Morvan fibrillary chorea;
119-2	A	Anti-VGKC antibody-associated limbic encephalitis;
120	A	Hereditary dystonia;
120-1	A	DYT1 dystonia;
120-2	A	DYT2 dystonia;
120-3	A	DYT3 dystonia;X-linked dystonia-parkinsonism;Lubag;
120-4	A	DYT4 dystonia;
120-5	A	DYT5 dystonia;Segawa syndrome;Dopa-responsive dystonia;
120-6	A	DYT6 dystonia;
120-7	A	DYT7 dystonia;
120-8	A	DYT8 dystonia;Paroxysmal nonkinesigenic dyskinesia 1;PNKD1;
120-9	A	DYT9 dystonia;Paroxysmal dystonic choreathetosis with episodic ataxia and spasticity;Paroxysmal choreoathetosis and episodic ataxia and spasticity;
120-10	A	DYT10 dystonia;Episodic kinesigenic dyskinesia 1;EKD1;
120-11	A	DYT11 dystonia;Myoclonus-dystonia syndrome;
120-12	A	DYT12 dystonia;Rapid-onset dystonia-parkinsonism;Alternating hemiplegia of childhood;Cerebellar ataxia, areflexia, pes cavus, optic atropy, and sensorineural hearing loss;CAPOS;
120-13	A	DYT13 dystonia;
120-14	A	DYT14 dystonia;Segawa syndrome;Dopa-responsive dystonia;
120-15	A	DYT15 dystonia;
120-16	A	DYT16 dystonia;
120-17	A	DYT17 dystonia;
120-18	A	DYT18 dystonia;Paroxysmal execise-induced dyskinesia;
120-19	A	DYT19 dystonia;Episodic kinesigenic dyskinesia 2;
120-20	A	DYT20 dystonia;Paroxysmal nonkinesigenic dyskinesia 2;PNKD2;
120-21	A	Neurodegeneration with Brain Iron Accumulation 1;Pantothenate kinase-associated neurodegeneration;PKAN;NBIA1;Hallervorden-Spatz syndrome;Hypoprebetalipoproteinemia, acanthocytosis, retinitis pigmentosa, pallidal degeneration;
120-22	A	Neurodegeneration with Brain Iron Accumulation 2;Infantile neuroaxonal dystrophy;INAD;NBIA2;Karak syndrome;
120-23	A	Neurodegeneration with Brain Iron Accumulation 3;Neuroferritinopathy;NBIA3;

120-24	A	Neurodegeneration with Brain Iron Accumulation 4;Aceruloplasminemia;Hereditary ceruloplasmin deficiency;NBIA4;
120-25	A	Neurodegeneration with Brain Iron Accumulation 5;NBIA5;Beta-propeller protein-associated neurodegeneration;BPAN;
120-26	A	Fatty Acid Hydroxylase-associated neurodegeneration;Dysmyelinating leukodystrophy and spastic paraparasis with or without dystonia, spastic paraplegia 35;
121	A	Neuroferritinopathy;
122	A	Superficial siderosis;Brain table hemosiderosis;
123	A	Cerebral autosomal recessive arteriopathy with subcortical infarcts and leukoencephalopathy;Cerebral autosomal recessive arteriopathy with baldness and degenerative spondylosis;Autosomal recessive leukoencephalopathy with baldness and degenerative spondylosis;Cerebral autosomal recessive arteriopathy;Autosomal recessive leukoencephalopathy;
124	A	Cerebral autosomal dominant arteriopathy with subcortical infarcts and leukoencephalopathy;Autosomal dominant cerebral artery disease with subcortical infarct and leukoencephalopathy;Autosomal dominant cerebral artery disease;
125	A	Hereditary diffuse leukoencephalopathy with spheroid;Hereditary diffuse leukoencephalopathy;
126	A	Perry syndrome;
127	A	Frontotemporal lobar degeneration;
127-1	A	Frontotemporal dementia;
127-2	A	Semantic dementia;
128	A	Bickerstaff brainstem encephalitis;
129	A	Acute encephalopathy with biphasic seizures and late reduced diffusion;Epilepticus type biphasic acute encphalopathy;Epilepticus type acute encphalopathy;
130	A	Congenital insensitivity to pain with anhydrosis;CIPA;
130-1	A	Hereditary sensory and autonomic neuropathy type IV;HSAN4;
130-2	A	Hereditary sensory and autonomic neuropathy type V;HSAN5;
131	A	Alexander disease;
132	A	Congenital supranuclear bulbar palsy;Congenital suprabulbar paresis;Worcester drought syndrome;Worster-Drought syndrome;
133	A	Moebius syndrome;Mobius syndrome;
134	K	Septo-optic dysplasia;De Morsier syndrome;
135	A	Aicardi syndrome;
136	A	Hemimegalencephaly;Unilateral megalencephaly;
137	A	Focal cortical dysplasia;
138	A	Nerve cell migration disorder;Lissencephaly;Neuronal migration defect;
138-1	A	Classical Lissencephaly;
138-2	A	Ectopic gray matter;
138-3	A	Subcortical ectopic gray matter;
138-4	A	Periventricular nodular ectopic gray matter;
138-5	A	Polymicrogyria;
138-6	A	Cortical dysplasia with cobblestone appearance;
138-7	A	Schizencephaly;
138-8	A	Porencephaly;
138-9	A	Miller-Dieker syndrome;
138-10	A	Perisylvian polymicrogyria;
138-11	A	X-linked Lissencephaly;
139	A	Congenital cerebral hypomyelination;Congenital cerebral white matter aplasia;
139-1	A	Pelizaeus-Merzbacher disease;
139-2	A	Pelizaeus-Merzbacher-like disease 1;Pelizaeus-Merzbacher-like disease type 1;
139-3	A	Hypomyelination with atrophy of the basal ganglia and cerebellum;
139-4	A	18q-syndrome;Chromosome 18q deletion syndrome;
139-5	A	Allan-Herndon-Dudley syndrome;
139-6	A	Mitochondrial Hsp60 chaperonopathy;
139-7	A	Salla disease;
139-8	A	Diffuse cerebral hypomyelination with cerebellar atrophy and hypoplasia of the corpus callosum;
139-9	A	Hypomyelination and congenital cataract;
139-10	A	Ataxia, delayed dentition, and hypomyelination;
139-11	A	Peripheral demyelinating neuropathy;
139-12	A	Central dysmyelinating leukodystrophy;
139-13	A	Waardenburg syndrome;
139-14	A	Hirschsprung disease;
140	A	Dorabe syndrome;Dravet syndrome;
141	A	Mesial temporal lobe epilepsy with hippocampal sclerosis;Medial temporal lobe epilepsy with hippocampal sclerosis;Mesial temporal lobe epilepsy with bilateral hippocampal sclerosis;Medial temporal lobe epilepsy;
142	A	Myoclonic absence epilepsy;
143	A	Epilepsy with myoclonic-atonic seizure;Epilepsy with myoclonic cataplexy;
144	A	Lennox-Gastaut syndrome;
145	A	West syndrome;Infantile spasm;
146	A	Ohtahara syndrome;Early infantile epileptic encephalopathy with suppression burst;
147	A	Early myoclonic encephalopathy;
148	A	Epilepsy of infancy with migrating focal seizure;Infant epilepsy with migratory focus seizure;Migrating partial seizures in infancy;Infant epilepsy;
149	A	Hemiconvulsion hemiplegia epilepsy syndrome;One side convulsions;Hemiplegia;Epilepsy syndrome;
150	A	Ring chromosome 20 epilepsy syndrome;Ring chromosome 20 syndrome;

151	A	Rasmussen encephalitis;
152	A	PCDH19 related syndrome;PCDH19 Epilepsy;Epilepsy and mental retardation limited to females;PCDH19 female pediatric epilepsy;PCDH19-related epilepsy;Protocadherin 19 (PCDH19)-related epilepsy;
153	A	Acute encephalitis with refractory, repetitive partial seizure;AERRPS;Refractory frequent partial seizures intussusception acute encephalitis;Febrile infection related epilepsy syndrome;FIRES;New onset refractory status epilepsy syndrome;NORSE syndrome;
154	A	Epilepsy with continuous spikes and waves during slow sleep;Epileptic encephalopathy with continuous spike-and-wave during sleep;
155	A	Acquired aphasia with convulsive disorder;Landau-Kleffner syndrome;
156	A	Rett syndrome;
157	A	Sturge-Weber syndrome;Síndrome de Sturge-Weber;
158	A	Tuberous sclerosis;Tuberous sclerosis complex;
159	A	Xeroderma pigmentosum;
160	C	Congenital ichthyosis;
160-1	C	Keratinopathic ichthyosis;
160-2	C	Epidermolytic ichthyosis;
160-3	C	Superficial epidermolytic ichthyosis;
160-4	C	Harlequin ichthyosis;
160-5	C	Autosomal recessive congenital ichthyosis;
160-6	C	Congenital Ichthyosiform Erythroderma;
160-7	C	Foliate ichthyosis;
160-8	C	Ichthyosis syndrome;
160-9	C	Netherton syndrome;
160-10	C	Sjogren-Larsson syndrome;Sjögren-Larsson syndrome;
160-11	C	Keratitis-ichtyosis-deafness syndrome;
160-12	C	Dorfman-Chanarin syndrome;
160-13	C	Neutral lipid storage disease;NLSD;
160-14	C	Multiple sulfatase deficiency;Austin disease;
160-15	C	Recessive X-linked ichthyosis;RXLI;X-linked recessive ichthyosis;
160-16	C	Ichthyosis, brittle hair, impaired intelligence, decreased fertility and short stature;IBID;
160-17	C	Trichothiodystrophy;
160-18	C	Follicular ichthyosis;
160-19	C	Congenital hemidysplasia, ichthyosiform erythroderma or nevus, and limb defects syndrome;CHILD syndrome;
160-20	C	Conradi-Hunermann-Happle syndrome;Conradi-Hu¨nermann-Happle syndrome;
161	C	Familial benign chronic pemphigus;Benign familial pemphigus;Hailey-Hailey disease;
162	C	Pemphigoid;
162-1	C	Epidermolysis bullosa acquisita;
163	C	Idiopathic pure sudomotor failure;Idiopathic acquired systemic anhidrosis;Acquired idiopathic generalized anhidrosis;AIGA;
163-1	C	Idiopathic segmental anhidrosis;
163-2	C	Idiopathic pure sudomotor failure;IPSF;
163-3	C	Sweat gland failure;
164	K	Oculocutaneous albinism;
164-1	K	Hermansky-Pudlak syndrome;
164-2	K	Chediak-Higashi syndrome;
164-3	K	Griscelli syndrome;
165	N	Pachydermoperiostosis;
166	C	Pseudoxanthoma elasticum;
167	C	Marfan syndrome;
168	C	Ehlers-Danlos syndrome;
169	B	Menkes disease;
170	C	Occipital horn syndrome;
171	B	Wilson disease;
172	H	Hypophosphatasia;
173	N	VATER syndrome;VATER association;VACTERL association;
174	N	Nasu-Hakola disease;Polycystic lipomembranous osteodysplasia with sclerosing leukoencephalopathy;PLOSL;
175	N	Weaver syndrome;
176	N	Coffin-Lowry syndrome;
177	A	Joubert syndrome related disorder;Joubert syndrome and related disorder;Joubert syndrome;JSRD;
177-1	A	Arima syndrome;
177-2	A	Senior-Loken syndrome;Senior-Løken syndrome;
177-3	A	COACH syndrome;
177-4	A	Orofaciodigital syndrome;
178	N	Mowat-Wilson syndrome;
179	N	Williams syndrome;
180	N	ATR-X syndrome;Alpha-thalassemia mental retardation syndrome;
181	N	Crouzon syndrome;
182	N	Apert syndrome;
183	N	Pfeiffer syndrome;
184	N	Antley-Bixler syndrome;
185	N	Coffin-Siris syndrome;

186	N	Rothmund-Thomson syndrome;
186-1	N	RAPADILINO syndrome;
186-2	N	Baller-Gerold syndrome;
187	N	Kabuki syndrome;
188	N	Polysplenia syndrome;
189	N	Asplenia syndrome;
190	L	Branchio-oto-renal syndrome;BOR syndrome;
191	N	Werner syndrome;
192	N	Cockayne syndrome;
193	N	Prader-Willi syndrome;
194	N	Sotos syndrome;
195	N	Noonan syndrome;
196	N	Young-Simpson syndrome;
197	N	1p36 deletion syndrome;
198	N	4p deletion syndrome;4p-syndrome;
199	N	5p deletion syndrome;5p-syndrome;
200	N	Paternal uniparental disomy of chromosome 14;No. 14 chromosome father disomy syndrome;Kagami-Ogata syndrome;
201	A	Angelman syndrome;
202	N	Smith-Magenis syndrome;
203	N	22q11.2 deletion syndrome;
204	N	Emanuel syndrome;Derivative 22 syndrome;
205	N	Fragile X syndrome related disease;Fragile X-associated tremor/ataxia syndrome;FXTAS;
206	N	Fragile X syndrome;
207	E	Persistent truncus arteriosus;
208	E	Corrected transposition of great arteries;
209	E	Complete transposition of great vessel;Complete transposition of great arteries;
210	E	Single ventricle heart defect;Complete TGA;Univentricular heart;Single ventricular circulation syndrome;
211	E	Hypoplastic left heart syndrome;Single ventricular circulation syndrome;
212	E	Tricuspid atresia;Single ventricular circulation syndrome;
213	E	Pulmonary atresia without ventricular septum defect;Pulmonary atresia with intact ventricular septum;Pulmonary atresia;Single ventricular circulation syndrome;
214	E	Pulmonary atresia with ventricular septum defect;Pulmonary atresia with ventricular septal defect;Pulmonary atresia;Tetralogy of Fallot with severe pulmonary stenosis;
215	E	Tetralogy of Fallot;Fallot tetralogy;
216	E	Double outlet right ventricle;
217	E	Ebstein disease;Ebstein malformation;
218	G	Alport syndrome;
219	G	Galloway-Mowat syndrome;
220	G	Rapidly progressive glomerulonephritis;
221	G	Anti-glomerular basement membrane disease;
222	G	Primary nephrotic syndrome;
222-1	G	Minimal change nephrotic syndrome;MCNS;
222-2	G	Membranous nephropathy;
222-3	G	Focal segmental glomerulosclerosis;FSGS;
222-4	G	Membranoproliferative glomerulonephritis;MPGN;
223	G	Primary membranoproliferative glomerulonephritis;
223-1	G	Dense deposit disease;
224	G	Purpura nephritis;
225	G	Congenital nephrogenic diabetes insipidus;Hereditary nephrogenic diabetes insipidus;Nephrogenic diabetes insipidus;
226	G	Interstitial cystitis with Hunners ulcer;Interstitial cystitis;
227	N	Osler disease;Hereditary hemorrhagic telangiectasia;Osler-Weber-Rendu disease;
228	J	Bronchiolitis obliterans;Obliterating bronchiolitis;
229	J	Autoimmune pulmonary alveolar proteinosis;Congenital pulmonary alveolar proteinosis;Hereditary pulmonary alveolar proteinosis;Pulmonary alveolar proteinosis;
229-1	J	Autoimmune pulmonary alveolar proteinosis;
229-2	J	Congenital pulmonary alveolar proteinosis;Hereditary pulmonary alveolar proteinosis;
230	J	Alveolar hypoventilation syndrome;Hypoventilation syndrome;
231	J	Alpha-1-antitrypsin deficiency;AATD;
232	N	Carney complex;
233	I	Wolfram syndrome;Diabetes Insipidus, Diabetes mellitus, optic atrophy, and deafness syndrome;DIDMOAD syndrome;
234	B	Peroxisomal disease (except Adrenoleukodystrophy);Peroxisomal disease;Peroxisomal disorder;Peroxisome biogenesis disorder;Zellweger syndrome;
234-1	B	Peroxisome biogenesis disorder;PEX gene disorder;
234-2	B	Zellweger syndrome;
234-3	B	Neonatal adrenoleukodystrophy;
234-4	B	Infantile Refsum disease;
234-5	B	Rhizomelic chondrodysplasia punctata type 1;RCDP type 1;RCDP1;
234-6	B	Peroxisomal beta-oxidation enzyme deficiency;
234-7	B	Acyl-CoA oxidase deficiency;AOX deficiency;

234-8	B	D-Bifunctional protein deficiency;DBP deficiency;
234-9	B	Sterol carrier protein X deficiency;SCPx deficiency;
234-10	B	2-methylacyl-CoA racemase deficiency;Alpha-methylacyl-CoA racemase deficiency;AMACR deficiency;
234-11	B	Refsum disease;
234-12	B	Plasmalogen biosynthesis enzyme deficiency;
234-13	B	Rhizomelic chondrodysplasia punctata type 2;RCDP type 2;RCDP2;
234-14	B	Rhizomelic chondrodysplasia punctata type 3;RCDP type 3;RCDP3;
234-15	B	Primary hyperoxaluria type 1;
234-16	B	Acatalasemia;Acatalasia;
234-17	B	Contiguous ABCD1/DXS1357E deletion syndrome;CADDS;
235	I	Hypoparathyroidism;Accessory thyroid hypergasia disease;
236	I	Pseudohypoparathyroidism;
237	I	ACTH unresponsiveness;Adrenocorticotropic hormone unresponsiveness;Adrenocorticotropic hormone insensitivity;
237-1	I	Triple A syndrome;Allgrove syndrome;
238	H	Vitamin D-resistant rickets;Vitamin D-resistant osteomalacia;VDRR;FGF23-related hypophosphatemic disease;FGF23-related hypophosphatemia;
239	I	Vitamin D-dependent rickets;Vitamin D-dependent osteomalacia;VDDR;
240	B	Phenylketonuria;
240-1	B	Phenylalanine hydroxylase deficiency;PAH deficiency;
240-2	B	Tetrahydrobiopterin deficiency;BH4 deficiency;
240-3	B	BH4 reactive hyper pheemia;
241	B	Hypertyrosinemia type I;Tyrosinemia type I;Tyrosinemia I;Hereditary tyrosinemia, Type I;
242	B	Hypertyrosinemia type II;Tyrosinemia type II;Tyrosinemia II;Hereditary tyrosinemia, Type II;
243	B	Hypertyrosinemia type III;High tyrosinemia;Tyrosinemia type III;Tyrosinemia III;Hereditary tyrosinemia, Type III;
244	B	Maple syrup urine disease;MSUD;
245	B	Propionic acidemia;
246	B	Methylmalonic acidemia;
247	B	Isovaleric acidemia;Isovaleric aciduria;Isovaleric acid CoA dehydrogenase deficiency;
248	B	Glucose transporter type 1 deficiency;GLUT1 deficiency;
249	B	Glutaric acidemia type 1;
250	B	Glutaric acidemia type 2;Multiple acyl-CoA dehydrogenase deficiency;Multiple acyl-CoA dehydrogenation deficiency;MADD;
251	B	Urea cycle disorder;
251-1	B	N-acetylglutamate synthase deficiency;NAGS deficiency;
251-2	B	Carbamoyl phosphate synthetase I deficiency;CPS1 deficiency;
251-3	B	Ornithine transcarbamylase deficiency;OTC deficiency;
251-4	B	Classic citrullinemia;Citrullinemia type I;
251-5	B	Argininosuccinic aciduria;
251-6	B	Argininemia;
251-7	B	Hiperornitinemia-hiperamonemia-homocitrulinuria syndrome;HHH syndrome;
252	B	Lysinuric protein intolerance;
253	B	Congenital folate malabsorption;Hereditary folate malabsorption;Folate malabsorption;
254	B	Porphyria;
254-1	B	Acute intermittent porphyria;
254-2	B	Hereditary coproporphyria;
254-3	B	Variegate porphyria;
254-4	B	Erythropoietic protoporphyria;
254-5	B	Porphyria cutanea tarda;
254-6	B	Congenital erythropoietic porphyria;
254-7	B	X-linked dominant protoporphyria;
254-8	B	Hepatoerythropoietic porphyria;
255	B	Multiple carboxylase deficiency;
255-1	B	Holocarboxylase synthetase deficiency;HCS deficiency;
255-2	B	Biotinidase deficiency;
256	B	Muscle glycogenosis;Muscular glycogenosis;Muscle glycogen storage disease;Muscular glycogen storage disease;
256-1	B	Glycogen storage disease type 0;GSD0;Glycogen synthase deficiency;
256-2	B	Glycogen storage disease type II;GSDII;Pompe disease;Alpha-1,4-glucosidase acid deficiency;
256-3	B	Glycogen storage disease type III;GSDIII;Cori disease;Glycogen debranching enzyme deficiency;
256-4	B	Glycogen storage disease type IV;GSDIV;Andersen disease;Glycogen-branching enzyme deficiency;GBED;
256-5	B	Glycogen storage disease type V;GSDV;McArdle disease;Muscle phosphorylase deficiency;Muscular phosphorylase deficiency;
256-6	B	Glycogen storage disease type VII;GSDVII;Tarui disease;Phosphofructokinase deficiency;PFK deficiency;
256-7	B	Glycogen storage disease type IX;GSDIX;Phosphorylase kinase deficiency;
256-8	B	Phosphoglycerate kinase deficiency;PGK deficiency;
256-9	B	Glycogen storage disease type X;GSDX;Phosphoglycerate mutase deficiency;
256-10	B	Glycogen storage diseass type XI;GSDXI;Kanno disease;Lactate dehydrogenase deficiency;
256-11	B	Glycogen storage diseass type XII;GSDXII;Aldolase A deficiency;
256-12	B	Glycogen storage diseass type XIII;GSDXIII;Beta-enolase deficiency;
256-13	B	Glycogen storage diseass type XIV;GSDXIV;Phosphoglucomutase deficiency;
256-14	B	Glycogen storage diseass type XV;GSDXV;Glycogenin 1 deficiency;
257	B	Hepatic glycogenosis;Liver glycogenosis;Hepatic glycogen storage disease;Liver glycogen storage disease;

257-1	B	Glycogen storage disease type I;GSDI;von Gierke disease;Glucose-6-phosphatase deficiency;G6Pase deficiency;
257-2	B	Glycogen storage disease type III;GSDIII;Cori disease;Glycogen debranching enzyme deficiency;
257-3	B	Glycogen storage disease type IV;GSDIV;Andersen disease;Glycogen-branching enzyme deficiency;GBED;Adult polyglucosan body disease;
257-4	B	Glycogen storage disease type VI;GSDVI;Hers disease;Hepatic phosphorylase deficiency;Liver phosphorylase deficiency;
257-5	B	Glycogen storage disease type IX;GSDIX;Phosphorylase kinase deficiency;
258	B	Galactose-1-phosphate uridylyltransferase deficiency;Galactose-1-phosphate uridyltransferase deficiency;Galactosemia type 1;GALT deficiency;
259	B	Lecithin-cholesterol acyltransferase deficiency;LCAT deficiency;
259-1	B	Fish-eye disease;
260	B	Sitosterolemia;
261	B	Tangier disease;
262	B	Primary hyperchylomicronemia;
263	B	Cerebrotendinous xanthomatosis;27-hydroxylase deficiency;CYP27 deficiency;
264	B	Abetalipoproteinemia;Microsomal triglyceride transfer protein deficiency;MTP deficiency;
265	B	Lipodystrophy;
265-1	B	Generalized congenital lipodystrophy;Berardinelli-Seip syndrome;
265-2	B	Familial partial lipodystrophy;Dunnigan-type familial partial lipodystrophy;Kobbering-type familial partial lipodystrophy;
265-3	B	Acquired generalized lipodystrophy;Lawrence syndrome;
265-4	B	Partial acquired lipodystrophy;Barraquer-Simons syndrome;
266	D	Familial mediterranean fever;
267	D	Hyper-IgD syndrome;Mevalonate kinase deffiency;Hyperimmunoglobulinemia D and periodic fever syndrome;
268	D	Nakajo-Nishimura syndrome;Autoinflammation, lipodystrophy, and dermatosis syndrome;CANDLE syndrome;JMP syndrome;Nakajo syndrome;
269	D	Pyogenic arthritis;Pyoderma gangrenosum;Acne syndrome;PAPA syndrome;
270	H	Chronic recurrent multifocal osteomyelitis;
271	H	Ankylosing spondylitis;Spondylarthritis ankylopoietica;
272	H	Fibrodysplasia ossificans progressiva;
273	H	Congenital scoliosis with rib anomaly;Congenital scoliosis;
274	H	Osteogenesis Imperfecta;
275	H	Thanatophoric dysplasia;
276	H	Achondroplasia;
277	J	Lymphangiomatosis;Generalized lymphatic anomaly;Gorham disease;Gorham-Stout disease;Diffuse lymphangiomatosis;Mass osteolysis;
278	J	Huge lymphatic malformation with cervicofacial lesion;Huge lymphatic malformation;Lymphatic malformation;
279	E	Huge venous malformation with cervical, oral and pharyngeal diffuse lesion;Huge venous malformation;Venous malformation;
280	E	Huge arteriovenous malformation with cervicofacial or limb lesion;Huge arteriovenous malformation;Arteriovenous malformation;
281	E	Klippel-Trenaunay-Weber syndrome;
282	F	Congenital dyserythropoietic anemia;
283	F	Acquired pure red cell aplasia;Pure red cell aplasia;
284	F	Diamond-Blackfan anemia;
285	F	Fanconi anemia;
286	F	Hereditary sideroblastic anemia;Congenital sideroblastic anemia;Sideroblastic anemia;
287	N	Epstein syndrome;
288	D	Autoimmune acquired coagulation factor deficiency;Coagulation factor deficiency;
288-1	D	Factor XIII deficiency;
288-2	D	Factor VIII deficiency;Acquired hemophilia A;
288-3	D	von Willebrand Disease;
288-4	D	Factor V deficiency;
289	M	Cronkhite-Canada syndrome;
290	M	Chronic nonspecific multiple ulcers of the small intestine;Nonspecific multiple ulcers in the small intestine;
291	M	Hirschsprung disease, entire colon type;Hirschsprung disease, small intestine type;Hirschsprung disease;Hirschprung disease;Hirschsprung disease associated entercolitis;
292	M	Cloacal exstrophy;Vesicointestinal fissure;
293	M	Persistent cloaca;
294	J	Congenital diaphragmatic hernia;
295	M	Infant huge hepatic hemangioma;Infant giant liver hemangioma;
296	M	Biliary atresia;
297	N	Alagille syndrome;
298	M	Hereditary pancreatitis;Chronic pancreatitis;
299	M	Cystic fibrosis;
300	D	IgG4-related disease;
300-1	D	Autoimmune pancreatitis;
300-2	D	IgG4-related sclerosing cholangitis;
300-3	D	IgG4-related lacrimal gland, orbital, and salivary gland lesions;
300-4	D	IgG4-related kidney disease;
301	K	Macular dystrophy;
301-1	K	Vitelliform macular dystrophy;Best vitelliform macular dystrophy;Best disease;
301-2	K	Stargardt disease;
301-3	K	Occult macular dystrophy;
301-4	K	Cone dystrophy;Cone rod dystrophy;
301-5	K	X-linked juvenile retinoschisis;

301-6	K	Central areolar choroidal dystrophy;
302	K	Leber hereditary optic neuropathy;
303	KO	Usher syndrome;
304	O	Juvenile-onset bilateral sensorineural hearing loss;
305	O	Delayed endolymphatic hydrops;
306	DO	Eosinophilic sinusitis;
307	A	Canavan disease;
308	A	Progressive leukoencephalopathy;
308-1	A	Megalencephalic leukoencephalopathy with subcortical cyst;
308-2	A	Leukoencephalopathy with vanishing white matter;
308-3	A	Leukoencephalopathy, progressive, with ovarian failure;
309	A	Progressive myoclonus epilepsy;
309-1	A	Unverricht-Lundborg disease;
309-2	A	Lafora disease;
309-3	A	Benign adult familial myoclonus epilepsy;BAFME;
310	N	Congenital anomalies syndrome;
310-1	N	Partial trisomy 1q syndrome;Trisomy 1q;
310-2	N	9q34 deletion syndrome;
310-3	N	Cornelia de Lange syndrome;CdLS;
310-4	N	Smith-Lemli-Opitz syndrome;SLO syndrome;
311	E	Congenital tricuspid stenosis;
312	E	Congenital mitral stenosis;
313	E	Congenital pulmonary vein stenosis;
314	E	Vascular sling;
315	G	Nail-Patella syndrome;LMX1B-associated nephropathy;
315-1	G	Nail-Patella syndrome;
315-2	G	LMX1B-associated nephropathy;
316	B	Carnitine cycle disorder;
316-1	B	Carnitine palmitoyltransferase I deficiency;CPT1 deficiency;
316-2	B	Carnitine palmitoyltransferase II deficiency;CPT2 deficiency;
316-3	B	Carnitine-acylcarnitine translocase deficiency;CACT deficiency;
316-4	B	Carnitine transporter deficiency;OCTN-2 deficiency;
317	B	Trifunctional protein deficiency;
318	B	Citrin deficiency;
318-1	B	Neonatal intrahepatic cholestasis caused by citrin deficiency;NICCD;
318-2	B	Adult-onset type II citrullinemia;CTLN2;
319	B	Sepiapterin reductase deficiency;
320	A	Inherited glycosylphosphatidylinositol deficiency;Congenital glycosylphosphatidylinositol deficiency;
321	B	Non-ketotic hyperglycinemia;
322	B	Beta-ketothiolase deficiency;
323	B	Aromatic L-amino acid decarboxylase deficiency;
324	B	Methylglutaconic aciduria;
324-1	B	3-methylglutaconyl-CoA hydratase deficiency;
324-2	B	Barth syndrome;
324-3	B	Costeff syndrome;
324-4	B	Mitochondrial respiratory chain disorder;
324-5	B	Dilated cardiomyopathy with ataxia syndrome;DCMA syndrome;
325	D	Hereditary autoinflammatory syndrome;
325-1	D	NLRC4 abnormality;
325-2	D	Adenosine deaminase 2 deficiency;ADA2 deficiency;
325-3	D	Aicardi-Goutieres syndrome;
325-4	D	A20 haploinsufficiency;
326	B	Osteopetrosis;
327	F	Idiopathic thrombosis;
328	K	Anterior segment dysgenesis;
329	K	Aniridia;
330	J	Congenital tracheal stenosis;Congenital subglottic stenosis;
330-1	J	Congenital tracheal stenosis;
330-2	J	Congenital subglottic stenosis;
331	F	Idiopathic multicentric castleman disease;Castleman disease;
332	K	Gelatinous drop-like corneal dystrophy;
333	N	Hutchinson-Gilford syndrome;Hutchinson-Gilford progeria syndrome;HGPS;

Supplementary Table 2. 753 drugs and 551 their target genes tested for rare and intractable diesases.

Accession number	Name	Target gene
DB00002	Cetuximab	EGFR
DB00005	Etanercept	LTA
DB00005	Etanercept	TNF
DB00007	Leuprolide	GNRHR
DB00014	Goserelin	GNRHR
DB00016	Erythropoietin	EPOR
DB00019	Pegfilgrastim	CSF3R
DB00020	Sargramostim	CSF2RA
DB00026	Anakinra	IL1R1
DB00026	Anakinra	IL1R2
DB00030	Insulin Human	INSR
DB00033	Interferon gamma-1b	IFNGR1
DB00033	Interferon gamma-1b	IFNGR2
DB00035	Desmopressin	AVPR2
DB00035	Desmopressin	F8
DB00038	Oprelvekin	IL11RA
DB00039	Palifermin	FGFR2
DB00040	Glucagon	GCGR
DB00041	Aldesleukin	IL2RA
DB00041	Aldesleukin	IL2RB
DB00041	Aldesleukin	IL2RG
DB00042	Botulinum Toxin Type B	SNAP25
DB00043	Omalizumab	FCER1A
DB00047	Insulin glargine	INSR
DB00050	Cetrorelix	GNRHR
DB00051	Adalimumab	TNF
DB00052	Somatotropin	GHR
DB00053	Imiglucerase	GBA
DB00060	Interferon beta-1a	IFNAR1
DB00060	Interferon beta-1a	IFNAR2
DB00065	Infliximab	TNF
DB00068	Interferon beta-1b	IFNAR1
DB00068	Interferon beta-1b	IFNAR2
DB00073	Rituximab	MS4A1
DB00074	Basiliximab	IL2RA
DB00075	Muromonab	CD3D
DB00075	Muromonab	CD3E
DB00075	Muromonab	CD3G
DB00082	Pegvisomant	GHR
DB00083	Botulinum toxin type A	SNAP25
DB00090	Laronidase	IDUA
DB00091	Ciclosporin	PPP3CA
DB00091	Ciclosporin	PPP3CB
DB00091	Ciclosporin	PPP3CC
DB00091	Ciclosporin	PPP3R1
DB00091	Ciclosporin	PPP3R2
DB00092	Alefacept	CD58
DB00095	Efalizumab	ITGAL
DB00099	Filgrastim	CSF3R
DB00103	Agalsidase beta	GLA
DB00104	Octreotide	SSTR2
DB00105	Interferon alfa-2b	IFNAR1
DB00105	Interferon alfa-2b	IFNAR2
DB00107	Oxytocin	OXTR
DB00108	Natalizumab	ITGA4
DB00111	Daclizumab	IL2RA
DB00112	Bevacizumab	VEGFA
DB00136	Calcitriol	VDR
DB00146	Calcifediol	VDR
DB00153	Ergocalciferol	VDR
DB00162	Vitamin A	RARA
DB00162	Vitamin A	RARB
DB00169	Cholecalciferol	VDR
DB00175	Pravastatin	HMGCR
DB00177	Valsartan	AGTR1
DB00178	Ramipril	ACE
DB00181	Baclofen	GABBR1
DB00181	Baclofen	GABBR2
DB00184	Nicotine	CHRNA1
DB00184	Nicotine	CHRNA10
DB00184	Nicotine	CHRNA2
DB00184	Nicotine	CHRNA3
DB00184	Nicotine	CHRNA4
DB00184	Nicotine	CHRNA5
DB00184	Nicotine	CHRNA6
DB00184	Nicotine	CHRNA7
DB00184	Nicotine	CHRNA9
DB00184	Nicotine	CHRNB1
DB00184	Nicotine	CHRNB2
DB00184	Nicotine	CHRNB3
DB00184	Nicotine	CHRNB4
DB00184	Nicotine	CHRND
DB00184	Nicotine	CHRNE
DB00184	Nicotine	CHRNG
DB00185	Cevimeline	CHRM3
DB00186	Lorazepam	GABRA1
DB00186	Lorazepam	GABRA2
DB00186	Lorazepam	GABRA3
DB00186	Lorazepam	GABRA4
DB00186	Lorazepam	GABRA5
DB00186	Lorazepam	GABRA6
DB00186	Lorazepam	GABRB1
DB00186	Lorazepam	GABRB2

DB00186	Lorazepam	GABRB3
DB00186	Lorazepam	GABRD
DB00186	Lorazepam	GABRE
DB00186	Lorazepam	GABRG1
DB00186	Lorazepam	GABRG2
DB00186	Lorazepam	GABRG3
DB00186	Lorazepam	GABRP
DB00186	Lorazepam	GABRQ
DB00188	Bortezomib	PSMB5
DB00190	Carbidopa	DDC
DB00193	Tramadol	OPRM1
DB00201	Caffeine	ADORA1
DB00201	Caffeine	ADORA2A
DB00201	Caffeine	PDE1B
DB00201	Caffeine	PDE2A
DB00201	Caffeine	PDE3A
DB00201	Caffeine	PDE3B
DB00201	Caffeine	PDE4A
DB00201	Caffeine	PDE4B
DB00201	Caffeine	PDE4C
DB00201	Caffeine	PDE4D
DB00201	Caffeine	PDE5A
DB00203	Sildenafil	PDE5A
DB00211	Midodrine	ADRA1A
DB00211	Midodrine	ADRA1B
DB00211	Midodrine	ADRA1D
DB00213	Pantoprazole	ATP4A
DB00213	Pantoprazole	ATP4B
DB00215	Citalopram	SLC6A4
DB00227	Lovastatin	HMGCR
DB00230	Pregabalin	CACNA2D1
DB00230	Pregabalin	CACNA2D2
DB00230	Pregabalin	CACNA2D3
DB00230	Pregabalin	CACNA2D4
DB00235	Milrinone	PDE3A
DB00235	Milrinone	PDE3B
DB00242	Cladribine	RRM1
DB00248	Cabergoline	DRD2
DB00248	Cabergoline	PRL
DB00252	Phenytoin	SCN1A
DB00252	Phenytoin	SCN2A
DB00252	Phenytoin	SCN3A
DB00252	Phenytoin	SCN5A
DB00252	Phenytoin	SCN9A
DB00264	Metoprolol	ADRB1
DB00268	Ropinirole	DRD2
DB00268	Ropinirole	DRD3
DB00268	Ropinirole	DRD4

DB00270	Isradipine	CACNA1C
DB00270	Isradipine	CACNA1D
DB00270	Isradipine	CACNA1F
DB00270	Isradipine	CACNA1S
DB00273	Topiramate	CA2
DB00273	Topiramate	CA4
DB00273	Topiramate	CACNA1C
DB00273	Topiramate	CACNA1D
DB00273	Topiramate	CACNA1F
DB00273	Topiramate	CACNA1S
DB00273	Topiramate	GABRA1
DB00273	Topiramate	GABRA2
DB00273	Topiramate	GABRA3
DB00273	Topiramate	GABRA4
DB00273	Topiramate	GABRA5
DB00273	Topiramate	GABRA6
DB00273	Topiramate	GABRB1
DB00273	Topiramate	GABRB2
DB00273	Topiramate	GABRB3
DB00273	Topiramate	GABRD
DB00273	Topiramate	GABRE
DB00273	Topiramate	GABRG1
DB00273	Topiramate	GABRG2
DB00273	Topiramate	GABRG3
DB00273	Topiramate	GABRP
DB00273	Topiramate	GABRQ
DB00273	Topiramate	GRIA1
DB00273	Topiramate	GRIA2
DB00273	Topiramate	GRIA3
DB00273	Topiramate	GRIA4
DB00273	Topiramate	GRIK1
DB00273	Topiramate	GRIK2
DB00273	Topiramate	GRIK3
DB00273	Topiramate	GRIK4
DB00273	Topiramate	GRIK5
DB00273	Topiramate	SCN1A
DB00273	Topiramate	SCN2A
DB00273	Topiramate	SCN3A
DB00273	Topiramate	SCN5A
DB00273	Topiramate	SCN9A
DB00277	Theophylline	ADORA1
DB00277	Theophylline	ADORA2A
DB00277	Theophylline	ADORA2B
DB00277	Theophylline	PDE10A
DB00277	Theophylline	PDE11A
DB00277	Theophylline	PDE1A
DB00277	Theophylline	PDE1B
DB00277	Theophylline	PDE1C

DB00277	Theophylline	PDE2A
DB00277	Theophylline	PDE3A
DB00277	Theophylline	PDE3B
DB00277	Theophylline	PDE4A
DB00277	Theophylline	PDE4B
DB00277	Theophylline	PDE4C
DB00277	Theophylline	PDE4D
DB00277	Theophylline	PDE7A
DB00277	Theophylline	PDE7B
DB00277	Theophylline	PDE8A
DB00277	Theophylline	PDE8B
DB00279	Liothyronine	THRA
DB00279	Liothyronine	THRB
DB00281	Lidocaine	SCN1A
DB00281	Lidocaine	SCN2A
DB00281	Lidocaine	SCN3A
DB00281	Lidocaine	SCN5A
DB00281	Lidocaine	SCN9A
DB00282	Pamidronic acid	FDPS
DB00283	Clemastine	HRH1
DB00285	Venlafaxine	SLC6A2
DB00285	Venlafaxine	SLC6A4
DB00289	Atomoxetine	SLC6A2
DB00295	Morphine	OPRM1
DB00296	Ropivacaine	SCN1A
DB00296	Ropivacaine	SCN2A
DB00296	Ropivacaine	SCN3A
DB00296	Ropivacaine	SCN5A
DB00296	Ropivacaine	SCN9A
DB00297	Bupivacaine	SCN1A
DB00297	Bupivacaine	SCN2A
DB00297	Bupivacaine	SCN3A
DB00297	Bupivacaine	SCN5A
DB00297	Bupivacaine	SCN9A
DB00302	Tranexamic acid	PLG
DB00304	Desogestrel	ESR1
DB00304	Desogestrel	PGR
DB00307	Bexarotene	RXRA
DB00307	Bexarotene	RXRB
DB00307	Bexarotene	RXRG
DB00313	Valproic Acid	ABAT
DB00313	Valproic Acid	ALDH5A1
DB00313	Valproic Acid	CACNA1G
DB00313	Valproic Acid	CACNA1H
DB00313	Valproic Acid	CACNA1I
DB00313	Valproic Acid	GAD1
DB00313	Valproic Acid	GAD2
DB00315	Zolmitriptan	HTR1B
DB00315	Zolmitriptan	HTR1D
DB00316	Acetaminophen	PTGS1
DB00316	Acetaminophen	PTGS2
DB00317	Gefitinib	EGFR
DB00321	Amitriptyline	SLC6A2
DB00321	Amitriptyline	SLC6A4
DB00323	Tolcapone	COMT
DB00328	Indometacin	PTGS1
DB00328	Indometacin	PTGS2
DB00331	Metformin	PRKAA1
DB00331	Metformin	PRKAA2
DB00332	Ipratropium	CHRM1
DB00332	Ipratropium	CHRM2
DB00332	Ipratropium	CHRM3
DB00332	Ipratropium	CHRM4
DB00332	Ipratropium	CHRM5
DB00334	Olanzapine	ADRA1A
DB00334	Olanzapine	ADRA1B
DB00334	Olanzapine	ADRA1D
DB00334	Olanzapine	DRD2
DB00334	Olanzapine	DRD3
DB00334	Olanzapine	DRD4
DB00334	Olanzapine	HRH1
DB00334	Olanzapine	HTR2A
DB00334	Olanzapine	HTR2B
DB00334	Olanzapine	HTR2C
DB00334	Olanzapine	HTR6
DB00335	Atenolol	ADRB1
DB00337	Pimecrolimus	PPP3CA
DB00337	Pimecrolimus	PPP3CB
DB00337	Pimecrolimus	PPP3CC
DB00337	Pimecrolimus	PPP3R1
DB00337	Pimecrolimus	PPP3R2
DB00338	Omeprazole	ATP4A
DB00338	Omeprazole	ATP4B
DB00341	Cetirizine	HRH1
DB00343	Diltiazem	CACNA1C
DB00343	Diltiazem	CACNA1D
DB00343	Diltiazem	CACNA1F
DB00343	Diltiazem	CACNA1S
DB00346	Alfuzosin	ADRA1A
DB00348	Nitisinone	HPD
DB00349	Clobazam	GABRA1
DB00349	Clobazam	GABRA2
DB00349	Clobazam	GABRA3
DB00349	Clobazam	GABRA4
DB00349	Clobazam	GABRA5
DB00349	Clobazam	GABRA6

DB00349	Clobazam	GABRB1
DB00349	Clobazam	GABRB2
DB00349	Clobazam	GABRB3
DB00349	Clobazam	GABRD
DB00349	Clobazam	GABRE
DB00349	Clobazam	GABRG1
DB00349	Clobazam	GABRG2
DB00349	Clobazam	GABRG3
DB00349	Clobazam	GABRP
DB00349	Clobazam	GABRQ
DB00350	Minoxidil	ABCC8
DB00350	Minoxidil	KCNJ11
DB00351	Megestrol acetate	PGR
DB00363	Clozapine	ADRA2A
DB00363	Clozapine	ADRA2B
DB00363	Clozapine	ADRA2C
DB00363	Clozapine	DRD4
DB00363	Clozapine	HTR2A
DB00363	Clozapine	HTR2C
DB00363	Clozapine	HTR3A
DB00363	Clozapine	HTR3B
DB00363	Clozapine	HTR3C
DB00363	Clozapine	HTR3D
DB00363	Clozapine	HTR3E
DB00367	Levonorgestrel	PGR
DB00373	Timolol	ADRB1
DB00373	Timolol	ADRB2
DB00374	Treprostinil	PTGIR
DB00376	Trihexyphenidyl	CHRM1
DB00376	Trihexyphenidyl	CHRM2
DB00376	Trihexyphenidyl	CHRM3
DB00376	Trihexyphenidyl	CHRM4
DB00376	Trihexyphenidyl	CHRM5
DB00378	Dydrogesterone	PGR
DB00379	Mexiletine	SCN1A
DB00379	Mexiletine	SCN2A
DB00379	Mexiletine	SCN3A
DB00379	Mexiletine	SCN5A
DB00379	Mexiletine	SCN9A
DB00380	Dexrazoxane	TOP2A
DB00380	Dexrazoxane	TOP2B
DB00381	Amlodipine	CACNA1C
DB00381	Amlodipine	CACNA1D
DB00381	Amlodipine	CACNA1F
DB00381	Amlodipine	CACNA1S
DB00390	Digoxin	ATP1A1
DB00390	Digoxin	ATP1A2
DB00390	Digoxin	ATP1A3
DB00390	Digoxin	ATP1A4
DB00394	Beclomethasone dipropionate	NR3C1
DB00398	Sorafenib	BRAF
DB00398	Sorafenib	FLT3
DB00398	Sorafenib	FLT4
DB00398	Sorafenib	KDR
DB00398	Sorafenib	KIT
DB00398	Sorafenib	PDGFRB
DB00398	Sorafenib	RAF1
DB00398	Sorafenib	RET
DB00399	Zoledronic acid	FDPS
DB00402	Eszopiclone	GABRA1
DB00402	Eszopiclone	GABRA2
DB00402	Eszopiclone	GABRA3
DB00402	Eszopiclone	GABRA4
DB00402	Eszopiclone	GABRA5
DB00402	Eszopiclone	GABRA6
DB00402	Eszopiclone	GABRB1
DB00402	Eszopiclone	GABRB2
DB00402	Eszopiclone	GABRB3
DB00402	Eszopiclone	GABRD
DB00402	Eszopiclone	GABRE
DB00402	Eszopiclone	GABRG1
DB00402	Eszopiclone	GABRG2
DB00402	Eszopiclone	GABRG3
DB00402	Eszopiclone	GABRP
DB00402	Eszopiclone	GABRQ
DB00404	Alprazolam	GABRA1
DB00404	Alprazolam	GABRA2
DB00404	Alprazolam	GABRA3
DB00404	Alprazolam	GABRA4
DB00404	Alprazolam	GABRA5
DB00404	Alprazolam	GABRA6
DB00404	Alprazolam	GABRB1
DB00404	Alprazolam	GABRB2
DB00404	Alprazolam	GABRB3
DB00404	Alprazolam	GABRD
DB00404	Alprazolam	GABRE
DB00404	Alprazolam	GABRG1
DB00404	Alprazolam	GABRG2
DB00404	Alprazolam	GABRG3
DB00404	Alprazolam	GABRP
DB00404	Alprazolam	GABRQ
DB00412	Rosiglitazone	PPARG
DB00413	Pramipexole	DRD2
DB00413	Pramipexole	DRD3
DB00413	Pramipexole	DRD4
DB00419	Miglustat	UGCG

DB00421	Spironolactone	NR3C2
DB00422	Methylphenidate	SLC6A2
DB00422	Methylphenidate	SLC6A3
DB00425	Zolpidem	GABRA1
DB00425	Zolpidem	GABRA2
DB00425	Zolpidem	GABRA3
DB00425	Zolpidem	GABRA4
DB00425	Zolpidem	GABRA5
DB00425	Zolpidem	GABRA6
DB00425	Zolpidem	GABRB1
DB00425	Zolpidem	GABRB2
DB00425	Zolpidem	GABRB3
DB00425	Zolpidem	GABRD
DB00425	Zolpidem	GABRE
DB00425	Zolpidem	GABRG1
DB00425	Zolpidem	GABRG2
DB00425	Zolpidem	GABRG3
DB00425	Zolpidem	GABRP
DB00425	Zolpidem	GABRQ
DB00437	Allopurinol	XDH
DB00443	Betamethasone	NR3C1
DB00448	Lansoprazole	ATP4A
DB00448	Lansoprazole	ATP4B
DB00449	Dipivefrin	ADRA1A
DB00449	Dipivefrin	ADRA1B
DB00449	Dipivefrin	ADRA1D
DB00449	Dipivefrin	ADRA2A
DB00449	Dipivefrin	ADRA2B
DB00449	Dipivefrin	ADRA2C
DB00449	Dipivefrin	ADRB1
DB00449	Dipivefrin	ADRB2
DB00449	Dipivefrin	ADRB3
DB00451	Levothyroxine	THRA
DB00451	Levothyroxine	THRB
DB00455	Loratadine	HRH1
DB00459	Acitretin	RARA
DB00459	Acitretin	RARB
DB00459	Acitretin	RXRA
DB00459	Acitretin	RXRB
DB00459	Acitretin	RXRG
DB00465	Ketorolac	PTGS1
DB00465	Ketorolac	PTGS2
DB00470	Dronabinol	CNR1
DB00470	Dronabinol	CNR2
DB00471	Montelukast	CYSLTR1
DB00472	Fluoxetine	SLC6A4
DB00476	Duloxetine	SLC6A2
DB00476	Duloxetine	SLC6A4
DB00477	Chlorpromazine	ADRA2A
DB00477	Chlorpromazine	ADRA2B
DB00477	Chlorpromazine	ADRA2C
DB00477	Chlorpromazine	CHRM1
DB00477	Chlorpromazine	CHRM2
DB00477	Chlorpromazine	CHRM3
DB00477	Chlorpromazine	CHRM4
DB00477	Chlorpromazine	CHRM5
DB00477	Chlorpromazine	DRD2
DB00477	Chlorpromazine	HRH1
DB00477	Chlorpromazine	HTR2A
DB00477	Chlorpromazine	HTR2B
DB00477	Chlorpromazine	HTR2C
DB00480	Lenalidomide	TNF
DB00481	Raloxifene	ESR1
DB00481	Raloxifene	ESR2
DB00482	Celecoxib	PTGS2
DB00484	Brimonidine	ADRA2A
DB00484	Brimonidine	ADRA2B
DB00484	Brimonidine	ADRA2C
DB00486	Nabilone	CNR1
DB00486	Nabilone	CNR2
DB00490	Buspirone	DRD2
DB00490	Buspirone	HTR1A
DB00494	Entacapone	COMT
DB00496	Darifenacin	CHRM3
DB00497	Oxycodone	OPRD1
DB00497	Oxycodone	OPRK1
DB00497	Oxycodone	OPRM1
DB00499	Flutamide	AR
DB00501	Cimetidine	HRH2
DB00502	Haloperidol	DRD2
DB00513	Aminocaproic Acid	PLG
DB00514	Dextromethorphan	GRIN1
DB00514	Dextromethorphan	GRIN2A
DB00514	Dextromethorphan	GRIN2B
DB00514	Dextromethorphan	GRIN2C
DB00514	Dextromethorphan	GRIN2D
DB00523	Alitretinoin	RARA
DB00523	Alitretinoin	RARB
DB00523	Alitretinoin	RXRA
DB00523	Alitretinoin	RXRB
DB00523	Alitretinoin	RXRG
DB00530	Erlotinib	EGFR
DB00540	Nortriptyline	SLC6A2
DB00540	Nortriptyline	SLC6A4
DB00541	Vincristine	TUBB
DB00541	Vincristine	TUBB1

DB00541	Vincristine	TUBB2A
DB00541	Vincristine	TUBB2B
DB00541	Vincristine	TUBB3
DB00541	Vincristine	TUBB4A
DB00541	Vincristine	TUBB4B
DB00541	Vincristine	TUBB6
DB00541	Vincristine	TUBB8
DB00542	Benazepril	ACE
DB00543	Amoxapine	SLC6A2
DB00543	Amoxapine	SLC6A4
DB00545	Pyridostigmine	ACHE
DB00555	Lamotrigine	SCN1A
DB00555	Lamotrigine	SCN2A
DB00555	Lamotrigine	SCN3A
DB00555	Lamotrigine	SCN5A
DB00555	Lamotrigine	SCN9A
DB00559	Bosentan	EDNRA
DB00559	Bosentan	EDNRB
DB00563	Methotrexate	DHFR
DB00563	Methotrexate	DHFR2
DB00564	Carbamazepine	SCN1A
DB00564	Carbamazepine	SCN2A
DB00564	Carbamazepine	SCN3A
DB00564	Carbamazepine	SCN5A
DB00564	Carbamazepine	SCN9A
DB00570	Vinblastine	TUBB
DB00570	Vinblastine	TUBB1
DB00570	Vinblastine	TUBB2A
DB00570	Vinblastine	TUBB2B
DB00570	Vinblastine	TUBB3
DB00570	Vinblastine	TUBB4A
DB00570	Vinblastine	TUBB4B
DB00570	Vinblastine	TUBB6
DB00570	Vinblastine	TUBB8
DB00571	Propranolol	ADRB1
DB00571	Propranolol	ADRB2
DB00571	Propranolol	ADRB3
DB00574	Fenfluramine	HTR2A
DB00574	Fenfluramine	HTR2B
DB00574	Fenfluramine	HTR2C
DB00574	Fenfluramine	SLC6A4
DB00575	Clonidine	ADRA2A
DB00575	Clonidine	ADRA2B
DB00575	Clonidine	ADRA2C
DB00580	Valdecoxib	PTGS2
DB00584	Enalapril	ACE
DB00586	Diclofenac	PTGS1
DB00586	Diclofenac	PTGS2

DB00588	Fluticasone propionate	NR3C1
DB00589	Lisuride	DRD2
DB00589	Lisuride	DRD3
DB00589	Lisuride	DRD4
DB00590	Doxazosin	ADRA1A
DB00590	Doxazosin	ADRA1B
DB00590	Doxazosin	ADRA1D
DB00591	Fluocinolone acetonide	NR3C1
DB00594	Amiloride	SCNN1A
DB00594	Amiloride	SCNN1B
DB00594	Amiloride	SCNN1G
DB00604	Cisapride	HTR2A
DB00604	Cisapride	HTR3A
DB00604	Cisapride	HTR4
DB00612	Bisoprolol	ADRB1
DB00619	Imatinib	ABL1
DB00619	Imatinib	KIT
DB00619	Imatinib	PDGFRA
DB00620	Triamcinolone	NR3C1
DB00621	Oxandrolone	AR
DB00622	Nicardipine	CACNA1C
DB00622	Nicardipine	CACNA1D
DB00622	Nicardipine	CACNA1F
DB00622	Nicardipine	CACNA1S
DB00624	Testosterone	AR
DB00629	Guanabenz	ADRA2A
DB00629	Guanabenz	ADRA2B
DB00629	Guanabenz	ADRA2C
DB00630	Alendronic acid	FDPS
DB00631	Clofarabine	POLA1
DB00631	Clofarabine	POLA2
DB00631	Clofarabine	RRM1
DB00633	Dexmedetomidine	ADRA2A
DB00633	Dexmedetomidine	ADRA2B
DB00633	Dexmedetomidine	ADRA2C
DB00635	Prednisone	NR3C1
DB00640	Adenosine	ADORA1
DB00640	Adenosine	ADORA2A
DB00640	Adenosine	ADORA2B
DB00640	Adenosine	ADORA3
DB00641	Simvastatin	HMGCR
DB00644	Gonadorelin	GNRHR
DB00659	Acamprosate	GRIN1
DB00659	Acamprosate	GRIN2A
DB00659	Acamprosate	GRIN2B
DB00659	Acamprosate	GRIN2C
DB00659	Acamprosate	GRIN2D
DB00661	Verapamil	CACNA1C

DB00661	Verapamil	CACNA1D
DB00661	Verapamil	CACNA1F
DB00661	Verapamil	CACNA1S
DB00668	Epinephrine	ADRA1A
DB00668	Epinephrine	ADRA1B
DB00668	Epinephrine	ADRA1D
DB00668	Epinephrine	ADRA2A
DB00668	Epinephrine	ADRA2B
DB00668	Epinephrine	ADRA2C
DB00668	Epinephrine	ADRB1
DB00668	Epinephrine	ADRB2
DB00668	Epinephrine	ADRB3
DB00674	Galantamine	ACHE
DB00675	Tamoxifen	ESR1
DB00675	Tamoxifen	ESR2
DB00678	Losartan	AGTR1
DB00682	Warfarin	NQO1
DB00682	Warfarin	VKORC1
DB00683	Midazolam	GABRA1
DB00683	Midazolam	GABRA2
DB00683	Midazolam	GABRA3
DB00683	Midazolam	GABRA4
DB00683	Midazolam	GABRA5
DB00683	Midazolam	GABRA6
DB00683	Midazolam	GABRB1
DB00683	Midazolam	GABRB2
DB00683	Midazolam	GABRB3
DB00683	Midazolam	GABRD
DB00683	Midazolam	GABRE
DB00683	Midazolam	GABRG1
DB00683	Midazolam	GABRG2
DB00683	Midazolam	GABRG3
DB00683	Midazolam	GABRP
DB00683	Midazolam	GABRQ
DB00687	Fludrocortisone	NR3C2
DB00688	Mycophenolate mofetil	IMPDH1
DB00688	Mycophenolate mofetil	IMPDH2
DB00691	Moexipril	ACE
DB00697	Tizanidine	ADRA2A
DB00697	Tizanidine	ADRA2B
DB00697	Tizanidine	ADRA2C
DB00700	Eplerenone	NR3C2
DB00704	Naltrexone	OPRD1
DB00704	Naltrexone	OPRK1
DB00704	Naltrexone	OPRM1
DB00708	Sufentanil	OPRM1
DB00710	Ibandronate	FDPS
DB00712	Flurbiprofen	PTGS1
DB00712	Flurbiprofen	PTGS2
DB00714	Apomorphine	DRD1
DB00714	Apomorphine	DRD2
DB00715	Paroxetine	SLC6A4
DB00717	Norethisterone	PGR
DB00722	Lisinopril	ACE
DB00724	Imiquimod	TLR7
DB00728	Rocuronium	CHRM1
DB00728	Rocuronium	CHRM2
DB00728	Rocuronium	CHRM3
DB00728	Rocuronium	CHRM4
DB00728	Rocuronium	CHRM5
DB00728	Rocuronium	CHRNA10
DB00728	Rocuronium	CHRNA2
DB00728	Rocuronium	CHRNA3
DB00728	Rocuronium	CHRNA4
DB00728	Rocuronium	CHRNA5
DB00728	Rocuronium	CHRNA6
DB00728	Rocuronium	CHRNA7
DB00728	Rocuronium	CHRNA9
DB00728	Rocuronium	CHRNB2
DB00728	Rocuronium	CHRNB3
DB00728	Rocuronium	CHRNB4
DB00734	Risperidone	DRD2
DB00734	Risperidone	HTR2A
DB00736	Esomeprazole	ATP4A
DB00736	Esomeprazole	ATP4B
DB00740	Riluzole	GRIA1
DB00740	Riluzole	GRIA2
DB00740	Riluzole	GRIA3
DB00740	Riluzole	GRIA4
DB00740	Riluzole	GRIK1
DB00740	Riluzole	GRIK2
DB00740	Riluzole	GRIK3
DB00740	Riluzole	GRIK4
DB00740	Riluzole	GRIK5
DB00740	Riluzole	GRIN1
DB00740	Riluzole	GRIN2A
DB00740	Riluzole	GRIN2B
DB00740	Riluzole	GRIN2C
DB00740	Riluzole	GRIN2D
DB00741	Hydrocortisone	NR3C1
DB00744	Zileuton	ALOX5
DB00747	Scopolamine	CHRM1
DB00747	Scopolamine	CHRM2
DB00747	Scopolamine	CHRM3
DB00747	Scopolamine	CHRM4
DB00747	Scopolamine	CHRM5

DB00749	Etodolac	PTGS2
DB00755	Tretinoin	PML
DB00755	Tretinoin	RARA
DB00755	Tretinoin	RARB
DB00758	Clopidogrel	P2RY12
DB00763	Methimazole	TPO
DB00770	Alprostadil	PTGER1
DB00773	Etoposide	TOP2A
DB00773	Etoposide	TOP2B
DB00776	Oxcarbazepine	SCN1A
DB00776	Oxcarbazepine	SCN2A
DB00776	Oxcarbazepine	SCN3A
DB00776	Oxcarbazepine	SCN5A
DB00776	Oxcarbazepine	SCN9A
DB00780	Phenelzine	MAOA
DB00780	Phenelzine	MAOB
DB00782	Propantheline	CHRM1
DB00782	Propantheline	CHRM2
DB00782	Propantheline	CHRM3
DB00782	Propantheline	CHRM4
DB00782	Propantheline	CHRM5
DB00783	Estradiol	ESR1
DB00783	Estradiol	ESR2
DB00788	Naproxen	PTGS1
DB00788	Naproxen	PTGS2
DB00790	Perindopril	ACE
DB00796	Candesartan cilexetil	AGTR1
DB00809	Tropicamide	CHRM1
DB00809	Tropicamide	CHRM2
DB00809	Tropicamide	CHRM3
DB00809	Tropicamide	CHRM4
DB00809	Tropicamide	CHRM5
DB00813	Fentanyl	OPRM1
DB00814	Meloxicam	PTGS2
DB00818	Propofol	GABRA1
DB00818	Propofol	GABRA2
DB00818	Propofol	GABRA3
DB00818	Propofol	GABRA4
DB00818	Propofol	GABRA5
DB00818	Propofol	GABRA6
DB00818	Propofol	GABRB1
DB00818	Propofol	GABRB2
DB00818	Propofol	GABRB3
DB00818	Propofol	GABRD
DB00818	Propofol	GABRE
DB00818	Propofol	GABRG1
DB00818	Propofol	GABRG2
DB00818	Propofol	GABRG3
DB00818	Propofol	GABRP
DB00818	Propofol	GABRQ
DB00819	Acetazolamide	CA1
DB00819	Acetazolamide	CA12
DB00819	Acetazolamide	CA13
DB00819	Acetazolamide	CA14
DB00819	Acetazolamide	CA2
DB00819	Acetazolamide	CA3
DB00819	Acetazolamide	CA4
DB00819	Acetazolamide	CA5A
DB00819	Acetazolamide	CA5B
DB00819	Acetazolamide	CA6
DB00819	Acetazolamide	CA7
DB00819	Acetazolamide	CA8
DB00819	Acetazolamide	CA9
DB00820	Tadalafil	PDE5A
DB00825	Levomenthol	TRPA1
DB00825	Levomenthol	TRPM8
DB00829	Diazepam	GABRA1
DB00829	Diazepam	GABRA2
DB00829	Diazepam	GABRA3
DB00829	Diazepam	GABRA4
DB00829	Diazepam	GABRA5
DB00829	Diazepam	GABRA6
DB00829	Diazepam	GABRB1
DB00829	Diazepam	GABRB2
DB00829	Diazepam	GABRB3
DB00829	Diazepam	GABRD
DB00829	Diazepam	GABRE
DB00829	Diazepam	GABRG1
DB00829	Diazepam	GABRG2
DB00829	Diazepam	GABRG3
DB00829	Diazepam	GABRP
DB00829	Diazepam	GABRQ
DB00831	Trifluoperazine	DRD2
DB00834	Mifepristone	NR3C1
DB00834	Mifepristone	PGR
DB00836	Loperamide	OPRD1
DB00836	Loperamide	OPRK1
DB00836	Loperamide	OPRM1
DB00841	Dobutamine	ADRB1
DB00843	Donepezil	ACHE
DB00844	Nalbuphine	OPRD1
DB00844	Nalbuphine	OPRK1
DB00844	Nalbuphine	OPRM1
DB00852	Pseudoephedrine	ADRA1A
DB00852	Pseudoephedrine	ADRA1B
DB00852	Pseudoephedrine	ADRA1D

DB00852	Pseudoephedrine	ADRA2A
DB00852	Pseudoephedrine	ADRA2B
DB00852	Pseudoephedrine	ADRA2C
DB00852	Pseudoephedrine	ADRB1
DB00852	Pseudoephedrine	ADRB2
DB00852	Pseudoephedrine	ADRB3
DB00860	Prednisolone	NR3C1
DB00861	Diflunisal	PTGS2
DB00862	Vardenafil	PDE5A
DB00863	Ranitidine	HRH2
DB00864	Tacrolimus	PPP3CA
DB00864	Tacrolimus	PPP3CB
DB00864	Tacrolimus	PPP3CC
DB00864	Tacrolimus	PPP3R1
DB00864	Tacrolimus	PPP3R2
DB00869	Dorzolamide	CA1
DB00869	Dorzolamide	CA12
DB00869	Dorzolamide	CA13
DB00869	Dorzolamide	CA14
DB00869	Dorzolamide	CA2
DB00869	Dorzolamide	CA3
DB00869	Dorzolamide	CA4
DB00869	Dorzolamide	CA5A
DB00869	Dorzolamide	CA5B
DB00869	Dorzolamide	CA6
DB00869	Dorzolamide	CA7
DB00869	Dorzolamide	CA8
DB00869	Dorzolamide	CA9
DB00872	Conivaptan	AVPR1A
DB00872	Conivaptan	AVPR2
DB00875	Flupentixol	DRD1
DB00875	Flupentixol	DRD2
DB00877	Sirolimus	MTOR
DB00882	Clomifene	ESR1
DB00882	Clomifene	ESR2
DB00884	Risedronic acid	FDPS
DB00887	Bumetanide	SLC12A2
DB00892	Oxybuprocaine	SCN1A
DB00892	Oxybuprocaine	SCN2A
DB00892	Oxybuprocaine	SCN3A
DB00892	Oxybuprocaine	SCN5A
DB00892	Oxybuprocaine	SCN9A
DB00899	Remifentanil	OPRM1
DB00904	Ondansetron	HTR3A
DB00904	Ondansetron	HTR3B
DB00904	Ondansetron	HTR3C
DB00904	Ondansetron	HTR3D
DB00904	Ondansetron	HTR3E
DB00908	Quinidine	SCN1A
DB00908	Quinidine	SCN2A
DB00908	Quinidine	SCN3A
DB00908	Quinidine	SCN5A
DB00908	Quinidine	SCN9A
DB00909	Zonisamide	CACNA1G
DB00909	Zonisamide	CACNA1H
DB00909	Zonisamide	CACNA1I
DB00909	Zonisamide	SCN1A
DB00909	Zonisamide	SCN2A
DB00909	Zonisamide	SCN3A
DB00909	Zonisamide	SCN5A
DB00909	Zonisamide	SCN9A
DB00910	Paricalcitol	VDR
DB00912	Repaglinide	ABCC8
DB00912	Repaglinide	KCNJ11
DB00915	Amantadine	GRIN1
DB00915	Amantadine	GRIN2A
DB00915	Amantadine	GRIN2B
DB00915	Amantadine	GRIN2C
DB00915	Amantadine	GRIN2D
DB00921	Buprenorphine	OPRK1
DB00921	Buprenorphine	OPRM1
DB00922	Levosimendan	PDE3A
DB00922	Levosimendan	PDE3B
DB00927	Famotidine	HRH2
DB00929	Misoprostol	PTGER2
DB00929	Misoprostol	PTGER3
DB00929	Misoprostol	PTGER4
DB00938	Salmeterol	ADRB2
DB00945	Acetylsalicylic acid	PTGS1
DB00945	Acetylsalicylic acid	PTGS2
DB00947	Fulvestrant	ESR1
DB00947	Fulvestrant	ESR2
DB00957	Norgestimate	PGR
DB00959	Methylprednisolone	NR3C1
DB00966	Telmisartan	AGTR1
DB00973	Ezetimibe	NPC1L1
DB00975	Dipyridamole	PDE4A
DB00975	Dipyridamole	PDE5A
DB00977	Ethinylestradiol	ESR1
DB00980	Ramelteon	MTNR1A
DB00980	Ramelteon	MTNR1B
DB00982	Isotretinoin	RARA
DB00982	Isotretinoin	RARB
DB00983	Formoterol	ADRB2
DB00986	Glycopyrronium	CHRM3
DB00988	Dopamine	DRD1

DB00988	Dopamine	DRD2
DB00988	Dopamine	DRD3
DB00988	Dopamine	DRD4
DB00988	Dopamine	DRD5
DB00989	Rivastigmine	ACHE
DB00996	Gabapentin	CACNA2D1
DB00996	Gabapentin	CACNA2D2
DB00996	Gabapentin	CACNA2D3
DB00996	Gabapentin	CACNA2D4
DB00996	Gabapentin	SLC6A1
DB00997	Doxorubicin	TOP2A
DB00997	Doxorubicin	TOP2B
DB01001	Salbutamol	ADRB2
DB01002	Levobupivacaine	SCN1A
DB01002	Levobupivacaine	SCN2A
DB01002	Levobupivacaine	SCN3A
DB01002	Levobupivacaine	SCN5A
DB01002	Levobupivacaine	SCN9A
DB01005	Hydroxyurea	RRM2
DB01006	Letrozole	CYP19A1
DB01009	Ketoprofen	PTGS1
DB01009	Ketoprofen	PTGS2
DB01011	Metyrapone	CYP11B1
DB01013	Clobetasol propionate	NR3C1
DB01018	Guanfacine	ADRA2A
DB01019	Bethanechol	CHRM1
DB01019	Bethanechol	CHRM2
DB01019	Bethanechol	CHRM3
DB01019	Bethanechol	CHRM4
DB01019	Bethanechol	CHRM5
DB01024	Mycophenolic acid	IMPDH1
DB01024	Mycophenolic acid	IMPDH2
DB01026	Ketoconazole	CYP17A1
DB01029	Irbesartan	AGTR1
DB01033	Mercaptopurine	HPRT1
DB01037	Selegiline	MAOB
DB01039	Fenofibrate	PPARA
DB01041	Thalidomide	TNF
DB01043	Memantine	GRIN1
DB01043	Memantine	GRIN2A
DB01043	Memantine	GRIN2B
DB01043	Memantine	GRIN2C
DB01043	Memantine	GRIN2D
DB01046	Lubiprostone	CLCN2
DB01049	Ergoloid mesylate	ADRA1A
DB01049	Ergoloid mesylate	ADRA1B
DB01049	Ergoloid mesylate	ADRA1D
DB01049	Ergoloid mesylate	ADRA2A
DB01049	Ergoloid mesylate	ADRA2B
DB01049	Ergoloid mesylate	ADRA2C
DB01050	Ibuprofen	PTGS1
DB01050	Ibuprofen	PTGS2
DB01062	Oxybutynin	CHRM1
DB01062	Oxybutynin	CHRM2
DB01062	Oxybutynin	CHRM3
DB01062	Oxybutynin	CHRM4
DB01062	Oxybutynin	CHRM5
DB01065	Melatonin	MTNR1A
DB01065	Melatonin	MTNR1B
DB01068	Clonazepam	GABRA1
DB01068	Clonazepam	GABRA2
DB01068	Clonazepam	GABRA3
DB01068	Clonazepam	GABRA4
DB01068	Clonazepam	GABRA5
DB01068	Clonazepam	GABRA6
DB01068	Clonazepam	GABRB1
DB01068	Clonazepam	GABRB2
DB01068	Clonazepam	GABRB3
DB01068	Clonazepam	GABRD
DB01068	Clonazepam	GABRE
DB01068	Clonazepam	GABRG1
DB01068	Clonazepam	GABRG2
DB01068	Clonazepam	GABRG3
DB01068	Clonazepam	GABRP
DB01068	Clonazepam	GABRQ
DB01069	Promethazine	HRH1
DB01070	Dihydrotachysterol	VDR
DB01073	Fludarabine	RRM1
DB01074	Perhexiline	CACNA1C
DB01074	Perhexiline	CACNA1D
DB01074	Perhexiline	CACNA1F
DB01074	Perhexiline	CACNA1S
DB01074	Perhexiline	CPT1A
DB01074	Perhexiline	CPT1B
DB01074	Perhexiline	CPT1C
DB01074	Perhexiline	CPT2
DB01075	Diphenhydramine	HRH1
DB01076	Atorvastatin	HMGCR
DB01080	Vigabatrin	ABAT
DB01085	Pilocarpine	CHRM2
DB01085	Pilocarpine	CHRM3
DB01088	Iloprost	PTGIR
DB01095	Fluvastatin	HMGCR
DB01097	Leflunomide	DHODH
DB01098	Rosuvastatin	HMGCR
DB01100	Pimozide	DRD2

DB01100	Pimozide	DRD3
DB01101	Capecitabine	TYMS
DB01104	Sertraline	SLC6A4
DB01109	Heparin	SERPINC1
DB01115	Nifedipine	CACNA1C
DB01115	Nifedipine	CACNA1D
DB01115	Nifedipine	CACNA1F
DB01115	Nifedipine	CACNA1S
DB01116	Trimethaphan	CHRNA10
DB01118	Amiodarone	ADRA1A
DB01118	Amiodarone	ADRA1B
DB01118	Amiodarone	ADRA1D
DB01118	Amiodarone	ADRB1
DB01118	Amiodarone	KCNJ11
DB01118	Amiodarone	KCNJ12
DB01118	Amiodarone	KCNJ3
DB01118	Amiodarone	KCNJ5
DB01118	Amiodarone	KCNJ8
DB01118	Amiodarone	KCNQ1
DB01118	Amiodarone	SCN1A
DB01118	Amiodarone	SCN2A
DB01118	Amiodarone	SCN3A
DB01118	Amiodarone	SCN5A
DB01118	Amiodarone	SCN9A
DB01119	Diazoxide	ABCC8
DB01119	Diazoxide	KCNJ11
DB01126	Dutasteride	SRD5A1
DB01126	Dutasteride	SRD5A2
DB01129	Rabeprazole	ATP4A
DB01129	Rabeprazole	ATP4B
DB01132	Pioglitazone	PPARG
DB01136	Carvedilol	ADRA1A
DB01136	Carvedilol	ADRA1B
DB01136	Carvedilol	ADRA1D
DB01136	Carvedilol	ADRB1
DB01136	Carvedilol	ADRB2
DB01142	Doxepin	HRH1
DB01142	Doxepin	SLC6A2
DB01142	Doxepin	SLC6A4
DB01144	Diclofenamide	CA1
DB01144	Diclofenamide	CA12
DB01144	Diclofenamide	CA13
DB01144	Diclofenamide	CA14
DB01144	Diclofenamide	CA2
DB01144	Diclofenamide	CA3
DB01144	Diclofenamide	CA4
DB01144	Diclofenamide	CA5A
DB01144	Diclofenamide	CA5B
DB01144	Diclofenamide	CA6
DB01144	Diclofenamide	CA7
DB01144	Diclofenamide	CA8
DB01144	Diclofenamide	CA9
DB01151	Desipramine	SLC6A2
DB01151	Desipramine	SLC6A4
DB01156	Bupropion	SLC6A2
DB01156	Bupropion	SLC6A3
DB01162	Terazosin	ADRA1A
DB01162	Terazosin	ADRA1B
DB01162	Terazosin	ADRA1D
DB01166	Cilostazol	PDE3A
DB01166	Cilostazol	PDE3B
DB01166	Cilostazol	TBXA2R
DB01171	Moclobemide	MAOA
DB01175	Escitalopram	SLC6A4
DB01183	Naloxone	OPRD1
DB01183	Naloxone	OPRK1
DB01183	Naloxone	OPRM1
DB01184	Domperidone	DRD2
DB01186	Pergolide	DRD1
DB01186	Pergolide	DRD2
DB01192	Oxymorphone	OPRM1
DB01195	Flecainide	SCN1A
DB01195	Flecainide	SCN2A
DB01195	Flecainide	SCN3A
DB01195	Flecainide	SCN5A
DB01195	Flecainide	SCN9A
DB01197	Captopril	ACE
DB01198	Zopiclone	GABRA1
DB01198	Zopiclone	GABRA2
DB01198	Zopiclone	GABRA3
DB01198	Zopiclone	GABRA4
DB01198	Zopiclone	GABRA5
DB01198	Zopiclone	GABRA6
DB01198	Zopiclone	GABRB1
DB01198	Zopiclone	GABRB2
DB01198	Zopiclone	GABRB3
DB01198	Zopiclone	GABRD
DB01198	Zopiclone	GABRE
DB01198	Zopiclone	GABRG1
DB01198	Zopiclone	GABRG2
DB01198	Zopiclone	GABRG3
DB01198	Zopiclone	GABRP
DB01198	Zopiclone	GABRQ
DB01200	Bromocriptine	DRD2
DB01200	Bromocriptine	PRL
DB01202	Levetiracetam	SV2A

DB01204	Mitoxantrone	TOP2A
DB01204	Mitoxantrone	TOP2B
DB01205	Flumazenil	GABRA1
DB01205	Flumazenil	GABRA2
DB01205	Flumazenil	GABRA3
DB01205	Flumazenil	GABRA4
DB01205	Flumazenil	GABRA5
DB01205	Flumazenil	GABRA6
DB01205	Flumazenil	GABRB1
DB01205	Flumazenil	GABRB2
DB01205	Flumazenil	GABRB3
DB01205	Flumazenil	GABRD
DB01205	Flumazenil	GABRE
DB01205	Flumazenil	GABRG1
DB01205	Flumazenil	GABRG2
DB01205	Flumazenil	GABRG3
DB01205	Flumazenil	GABRP
DB01205	Flumazenil	GABRQ
DB01213	Fomepizole	ADH1A
DB01213	Fomepizole	ADH1B
DB01213	Fomepizole	ADH1C
DB01216	Finasteride	SRD5A2
DB01217	Anastrozole	CYP19A1
DB01221	Ketamine	GRIN1
DB01221	Ketamine	GRIN2A
DB01221	Ketamine	GRIN2B
DB01221	Ketamine	GRIN2C
DB01221	Ketamine	GRIN2D
DB01222	Budesonide	NR3C1
DB01223	Aminophylline	ADORA1
DB01223	Aminophylline	ADORA2B
DB01223	Aminophylline	PDE10A
DB01223	Aminophylline	PDE11A
DB01223	Aminophylline	PDE1A
DB01223	Aminophylline	PDE1B
DB01223	Aminophylline	PDE1C
DB01223	Aminophylline	PDE2A
DB01223	Aminophylline	PDE3A
DB01223	Aminophylline	PDE3B
DB01223	Aminophylline	PDE4A
DB01223	Aminophylline	PDE4B
DB01223	Aminophylline	PDE4C
DB01223	Aminophylline	PDE4D
DB01223	Aminophylline	PDE7A
DB01223	Aminophylline	PDE7B
DB01223	Aminophylline	PDE8A
DB01223	Aminophylline	PDE8B
DB01224	Quetiapine	DRD2
DB01224	Quetiapine	HTR2A
DB01225	Enoxaparin	SERPINC1
DB01229	Paclitaxel	TUBB
DB01229	Paclitaxel	TUBB1
DB01229	Paclitaxel	TUBB2A
DB01229	Paclitaxel	TUBB2B
DB01229	Paclitaxel	TUBB3
DB01229	Paclitaxel	TUBB4A
DB01229	Paclitaxel	TUBB4B
DB01229	Paclitaxel	TUBB6
DB01229	Paclitaxel	TUBB8
DB01234	Dexamethasone	NR3C1
DB01235	Levodopa	DRD1
DB01235	Levodopa	DRD2
DB01235	Levodopa	DRD3
DB01235	Levodopa	DRD4
DB01235	Levodopa	DRD5
DB01238	Aripiprazole	DRD2
DB01238	Aripiprazole	DRD3
DB01238	Aripiprazole	HTR1A
DB01238	Aripiprazole	HTR2A
DB01240	Epoprostenol	PTGIR
DB01254	Dasatinib	ABL1
DB01254	Dasatinib	EPHA2
DB01254	Dasatinib	FYN
DB01254	Dasatinib	KIT
DB01254	Dasatinib	LCK
DB01254	Dasatinib	PDGFRB
DB01254	Dasatinib	SRC
DB01254	Dasatinib	YES1
DB01255	Lisdexamfetamine	SLC6A2
DB01255	Lisdexamfetamine	SLC6A3
DB01257	Eculizumab	C5
DB01259	Lapatinib	EGFR
DB01259	Lapatinib	ERBB2
DB01261	Sitagliptin	DPP4
DB01262	Decitabine	DNMT1
DB01262	Decitabine	DNMT3A
DB01262	Decitabine	DNMT3B
DB01268	Sunitinib	CSF1
DB01268	Sunitinib	FLT1
DB01268	Sunitinib	FLT3
DB01268	Sunitinib	FLT4
DB01268	Sunitinib	KDR
DB01268	Sunitinib	KIT
DB01268	Sunitinib	PDGFRA
DB01268	Sunitinib	PDGFRB
DB01268	Sunitinib	RET

DB01270	Ranibizumab	VEGFA
DB01271	Idursulfase	IDS
DB01272	Alglucosidase alfa	GAA
DB01273	Varenicline	CHRNA4
DB01273	Varenicline	CHRNB2
DB01275	Hydralazine	AOC3
DB01276	Exenatide	GLP1R
DB01277	Mecasermin	GHR
DB01277	Mecasermin	IGF1R
DB01279	Galsulfase	ARSB
DB01281	Abatacept	CD80
DB01281	Abatacept	CD86
DB01282	Carbetocin	OXTR
DB01283	Lumiracoxib	PTGS2
DB01284	Tetracosactide	MC2R
DB01285	Corticotropin	MC2R
DB01307	Insulin Detemir	INSR
DB01340	Cilazapril	ACE
DB01364	Ephedrine	ADRA1A
DB01364	Ephedrine	ADRA1B
DB01364	Ephedrine	ADRA1D
DB01364	Ephedrine	ADRA2A
DB01364	Ephedrine	ADRA2B
DB01364	Ephedrine	ADRA2C
DB01364	Ephedrine	ADRB1
DB01364	Ephedrine	ADRB2
DB01364	Ephedrine	ADRB3
DB01367	Rasagiline	MAOB
DB01380	Cortisone acetate	NR3C1
DB01393	Bezafibrate	PPARA
DB01394	Colchicine	TUBB
DB01394	Colchicine	TUBB1
DB01394	Colchicine	TUBB2A
DB01394	Colchicine	TUBB2B
DB01394	Colchicine	TUBB3
DB01394	Colchicine	TUBB4A
DB01394	Colchicine	TUBB4B
DB01394	Colchicine	TUBB6
DB01394	Colchicine	TUBB8
DB01396	Digitoxin	ATP1A1
DB01396	Digitoxin	ATP1A2
DB01396	Digitoxin	ATP1A3
DB01396	Digitoxin	ATP1A4
DB01399	Salsalate	PTGS1
DB01399	Salsalate	PTGS2
DB01406	Danazol	AR
DB01406	Danazol	ESR1
DB01406	Danazol	PGR

DB01407	Clenbuterol	ADRB2
DB01410	Ciclesonide	NR3C1
DB01436	Alfacalcidol	VDR
DB01586	Ursodeoxycholic acid	NR1H4
DB01590	Everolimus	MTOR
DB01591	Solifenacin	CHRM3
DB01628	Etoricoxib	PTGS2
DB01656	Roflumilast	PDE4A
DB01656	Roflumilast	PDE4B
DB01656	Roflumilast	PDE4C
DB01656	Roflumilast	PDE4D
DB01708	Prasterone	AR
DB01708	Prasterone	ESR1
DB01708	Prasterone	ESR2
DB01954	Rolipram	PDE4A
DB01954	Rolipram	PDE4B
DB01954	Rolipram	PDE4C
DB01954	Rolipram	PDE4D
DB02300	Calcipotriol	VDR
DB02546	Vorinostat	HDAC1
DB02546	Vorinostat	HDAC10
DB02546	Vorinostat	HDAC11
DB02546	Vorinostat	HDAC2
DB02546	Vorinostat	HDAC3
DB02546	Vorinostat	HDAC4
DB02546	Vorinostat	HDAC5
DB02546	Vorinostat	HDAC6
DB02546	Vorinostat	HDAC7
DB02546	Vorinostat	HDAC8
DB02546	Vorinostat	HDAC9
DB02659	Cholic Acid	NR1H4
DB03206	Duvoglustat	GAA
DB03404	Hemin	ALAS1
DB03404	Hemin	ALAS2
DB03424	Ubenimex	ANPEP
DB04573	Estriol	ESR1
DB04573	Estriol	ESR2
DB04630	Aldosterone	NR3C2
DB04743	Nimesulide	PTGS2
DB04818	Iproniazid	MAOA
DB04818	Iproniazid	MAOB
DB04835	Maraviroc	CCR5
DB04844	Tetrabenazine	SLC18A2
DB04846	Celiprolol	ADRB1
DB04847	Roxadustat	EGLN1
DB04847	Roxadustat	EGLN2
DB04847	Roxadustat	EGLN3
DB04849	Cediranib	FLT1

DB04849	Cediranib	FLT4
DB04849	Cediranib	KDR
DB04854	Febuxostat	XDH
DB04861	Nebivolol	ADRB1
DB04868	Nilotinib	ABL1
DB04868	Nilotinib	KIT
DB04868	Nilotinib	PDGFRA
DB04868	Nilotinib	PDGFRB
DB04896	Milnacipran	SLC6A2
DB04896	Milnacipran	SLC6A4
DB04912	Stannsoporfin	HMOX1
DB04912	Stannsoporfin	HMOX2
DB04942	Tamibarotene	RARA
DB04951	Pirfenidone	CCL2
DB04951	Pirfenidone	IL10
DB04951	Pirfenidone	IL1A
DB04951	Pirfenidone	IL1B
DB04951	Pirfenidone	IL6
DB04951	Pirfenidone	TNF
DB04953	Ezogabine	KCNQ1
DB04953	Ezogabine	KCNQ2
DB04953	Ezogabine	KCNQ3
DB04953	Ezogabine	KCNQ4
DB04953	Ezogabine	KCNQ5
DB04958	Epratuzumab	CD22
DB04960	Tipifarnib	FNTA
DB04960	Tipifarnib	FNTB
DB04982	Talampanel	GRIA1
DB04982	Talampanel	GRIA2
DB04982	Talampanel	GRIA3
DB04982	Talampanel	GRIA4
DB05076	Fenretinide	RARA
DB05076	Fenretinide	RARB
DB05087	Ganaxolone	GABRA1
DB05087	Ganaxolone	GABRA2
DB05087	Ganaxolone	GABRA3
DB05087	Ganaxolone	GABRA4
DB05087	Ganaxolone	GABRA5
DB05087	Ganaxolone	GABRA6
DB05087	Ganaxolone	GABRB1
DB05087	Ganaxolone	GABRB2
DB05087	Ganaxolone	GABRB3
DB05087	Ganaxolone	GABRD
DB05087	Ganaxolone	GABRE
DB05087	Ganaxolone	GABRG1
DB05087	Ganaxolone	GABRG2
DB05087	Ganaxolone	GABRG3
DB05087	Ganaxolone	GABRP
DB05087	Ganaxolone	GABRQ
DB05111	Fontolizumab	IFNG
DB05187	Elafibranor	PPARA
DB05229	Beraprost	PTGIR
DB05258	Interferon alfa	IFNAR1
DB05258	Interferon alfa	IFNAR2
DB05259	Glatiramer	HLA-A
DB05259	Glatiramer	HLA-B
DB05259	Glatiramer	HLA-C
DB05259	Glatiramer	HLA-DMA
DB05259	Glatiramer	HLA-DMB
DB05259	Glatiramer	HLA-DOA
DB05259	Glatiramer	HLA-DOB
DB05259	Glatiramer	HLA-DPA1
DB05259	Glatiramer	HLA-DPB1
DB05259	Glatiramer	HLA-DQA1
DB05259	Glatiramer	HLA-DQA2
DB05259	Glatiramer	HLA-DQB1
DB05259	Glatiramer	HLA-DRA
DB05259	Glatiramer	HLA-DRB1
DB05259	Glatiramer	HLA-DRB3
DB05259	Glatiramer	HLA-DRB4
DB05259	Glatiramer	HLA-DRB5
DB05259	Glatiramer	HLA-E
DB05259	Glatiramer	HLA-F
DB05259	Glatiramer	HLA-G
DB05266	Ibudilast	CYSLTR1
DB05266	Ibudilast	PDE3A
DB05266	Ibudilast	PDE3B
DB05266	Ibudilast	PDE4A
DB05266	Ibudilast	PDE4B
DB05266	Ibudilast	PDE4C
DB05266	Ibudilast	PDE4D
DB05266	Ibudilast	PTGIR
DB05271	Rotigotine	DRD2
DB05289	Tarenflurbil	APH1A
DB05289	Tarenflurbil	NCSTN
DB05289	Tarenflurbil	PSEN1
DB05289	Tarenflurbil	PSENEN
DB05295	Eldecalcitol	VDR
DB05332	Romiplostim	MPL
DB05351	Dexlansoprazole	ATP4A
DB05351	Dexlansoprazole	ATP4B
DB05386	Regramostim	CSF2RA
DB05394	Corticorelin	CRHR1
DB05394	Corticorelin	CRHR2
DB05496	Otelixizumab	CD3D
DB05496	Otelixizumab	CD3E

DB05496	Otelixizumab	CD3G
DB05541	Brivaracetam	SV2A
DB05656	Veltuzumab	MS4A1
DB05676	Apremilast	PDE4A
DB05676	Apremilast	PDE4B
DB05676	Apremilast	PDE4C
DB05676	Apremilast	PDE4D
DB05679	Ustekinumab	IL12A
DB05679	Ustekinumab	IL12B
DB05679	Ustekinumab	IL23A
DB05777	Thrombomodulin Alfa	F2
DB05829	Parathyroid hormone	PTH1R
DB05829	Parathyroid hormone	PTH2R
DB05990	Obeticholic acid	NR1H4
DB06014	Eteplirsen	DMD
DB06075	Linsitinib	IGF1R
DB06081	Caplacizumab	VWF
DB06155	Rimonabant	CNR1
DB06168	Canakinumab	IL1B
DB06193	Pixantrone	TOP2A
DB06193	Pixantrone	TOP2B
DB06201	Rufinamide	SCN1A
DB06201	Rufinamide	SCN2A
DB06201	Rufinamide	SCN3A
DB06201	Rufinamide	SCN5A
DB06201	Rufinamide	SCN9A
DB06207	Silodosin	ADRA1A
DB06212	Tolvaptan	AVPR2
DB06213	Regadenoson	ADORA2A
DB06218	Lacosamide	SCN3A
DB06218	Lacosamide	SCN9A
DB06228	Rivaroxaban	F10
DB06230	Nalmefene	OPRD1
DB06230	Nalmefene	OPRK1
DB06230	Nalmefene	OPRM1
DB06243	Eflornithine	ODC1
DB06262	Droxidopa	ADRA1A
DB06262	Droxidopa	ADRA1B
DB06262	Droxidopa	ADRA1D
DB06262	Droxidopa	ADRA2A
DB06262	Droxidopa	ADRA2B
DB06262	Droxidopa	ADRA2C
DB06262	Droxidopa	ADRB1
DB06262	Droxidopa	ADRB2
DB06262	Droxidopa	ADRB3
DB06267	Udenafil	PDE5A
DB06268	Sitaxentan	EDNRA
DB06271	Sulodexide	SERPINC1
DB06271	Sulodexide	SERPIND1
DB06273	Tocilizumab	IL6R
DB06285	Teriparatide	PTH1R
DB06287	Temsirolimus	MTOR
DB06335	Saxagliptin	DPP4
DB06372	Rilonacept	IL1A
DB06372	Rilonacept	IL1B
DB06399	Atacicept	TNFSF13
DB06399	Atacicept	TNFSF13B
DB06403	Ambrisentan	EDNRA
DB06413	Armodafinil	SLC6A3
DB06448	Lonafarnib	FNTA
DB06448	Lonafarnib	FNTB
DB06480	Prucalopride	HTR4
DB06554	Gaboxadol	GABRA1
DB06554	Gaboxadol	GABRA2
DB06554	Gaboxadol	GABRA3
DB06554	Gaboxadol	GABRA4
DB06554	Gaboxadol	GABRA5
DB06554	Gaboxadol	GABRA6
DB06554	Gaboxadol	GABRB1
DB06554	Gaboxadol	GABRB2
DB06554	Gaboxadol	GABRB3
DB06554	Gaboxadol	GABRD
DB06554	Gaboxadol	GABRE
DB06554	Gaboxadol	GABRG1
DB06554	Gaboxadol	GABRG2
DB06554	Gaboxadol	GABRG3
DB06554	Gaboxadol	GABRP
DB06554	Gaboxadol	GABRQ
DB06576	Baminercept	LTB
DB06594	Agomelatine	HTR2C
DB06594	Agomelatine	MTNR1A
DB06594	Agomelatine	MTNR1B
DB06602	Reslizumab	IL5
DB06605	Apixaban	F10
DB06611	Pegsunercept	TNF
DB06612	Mepolizumab	IL5
DB06616	Bosutinib	ABL1
DB06616	Bosutinib	SRC
DB06626	Axitinib	FLT1
DB06626	Axitinib	FLT4
DB06626	Axitinib	KDR
DB06637	Dalfampridine	KCNA4
DB06637	Dalfampridine	KCND2
DB06643	Denosumab	TNFSF11
DB06650	Ofatumumab	MS4A1
DB06654	Safinamide	MAOB

DB06655	Liraglutide	GLP1R
DB06663	Pasireotide	SSTR1
DB06663	Pasireotide	SSTR2
DB06663	Pasireotide	SSTR3
DB06663	Pasireotide	SSTR5
DB06666	Lixivaptan	AVPR2
DB06674	Golimumab	TNF
DB06681	Belatacept	CD80
DB06681	Belatacept	CD86
DB06702	Fesoterodine	CHRM2
DB06702	Fesoterodine	CHRM3
DB06712	Nilvadipine	CACNA1C
DB06712	Nilvadipine	CACNA1D
DB06712	Nilvadipine	CACNA1F
DB06712	Nilvadipine	CACNA1S
DB06718	Stanozolol	AR
DB06720	Velaglucerase alfa	GBA
DB06736	Aceclofenac	PTGES
DB06736	Aceclofenac	PTGS1
DB06736	Aceclofenac	PTGS2
DB06774	Capsaicin	TRPV1
DB06775	Carglumic acid	CPS1
DB06777	Chenodeoxycholic acid	NR1H4
DB06788	Histrelin	GNRHR
DB06809	Plerixafor	CXCR4
DB06825	Triptorelin	GNRHR
DB06826	Unoprostone	PTGFR
DB07138	Neflamapimod	MAPK11
DB07138	Neflamapimod	MAPK12
DB07138	Neflamapimod	MAPK13
DB07138	Neflamapimod	MAPK14
DB07425	Sobetirome	THRB
DB08162	Fasudil	ROCK1
DB08162	Fasudil	ROCK2
DB08439	Parecoxib	PTGS2
DB08804	Nandrolone decanoate	AR
DB08816	Ticagrelor	P2RY12
DB08820	Ivacaftor	CFTR
DB08827	Lomitapide	MTTP
DB08828	Vismodegib	SMO
DB08867	Ulipristal	PGR
DB08868	Fingolimod	S1PR1
DB08869	Tesamorelin	GHRHR
DB08870	Brentuximab vedotin	TNFRSF8
DB08870	Brentuximab vedotin	TUBB
DB08870	Brentuximab vedotin	TUBB1
DB08870	Brentuximab vedotin	TUBB2A
DB08870	Brentuximab vedotin	TUBB2B
DB08870	Brentuximab vedotin	TUBB3
DB08870	Brentuximab vedotin	TUBB4A
DB08870	Brentuximab vedotin	TUBB4B
DB08870	Brentuximab vedotin	TUBB6
DB08870	Brentuximab vedotin	TUBB8
DB08875	Cabozantinib	AXL
DB08875	Cabozantinib	FLT1
DB08875	Cabozantinib	FLT3
DB08875	Cabozantinib	FLT4
DB08875	Cabozantinib	KDR
DB08875	Cabozantinib	KIT
DB08875	Cabozantinib	MET
DB08875	Cabozantinib	NTRK2
DB08875	Cabozantinib	RET
DB08875	Cabozantinib	TEK
DB08876	Taliglucerase alfa	GBA
DB08877	Ruxolitinib	JAK1
DB08877	Ruxolitinib	JAK2
DB08879	Belimumab	TNFSF13B
DB08880	Teriflunomide	DHODH
DB08883	Perampanel	GRIA1
DB08883	Perampanel	GRIA2
DB08883	Perampanel	GRIA3
DB08883	Perampanel	GRIA4
DB08885	Aflibercept	PGF
DB08885	Aflibercept	VEGFA
DB08885	Aflibercept	VEGFB
DB08889	Carfilzomib	PSMB5
DB08890	Linaclotide	GUCY2C
DB08891	Arbaclofen	GABBR1
DB08891	Arbaclofen	GABBR2
DB08892	Arbaclofen Placarbil	GABBR1
DB08892	Arbaclofen Placarbil	GABBR2
DB08893	Mirabegron	ADRB3
DB08894	Peginesatide	EPOR
DB08895	Tofacitinib	JAK1
DB08895	Tofacitinib	JAK2
DB08895	Tofacitinib	JAK3
DB08895	Tofacitinib	TYK2
DB08900	Teduglutide	GLP2R
DB08904	Certolizumab pegol	TNF
DB08908	Dimethyl fumarate	KEAP1
DB08910	Pomalidomide	IL6
DB08910	Pomalidomide	TNF
DB08911	Trametinib	MAP2K1
DB08911	Trametinib	MAP2K2
DB08912	Dabrafenib	BRAF
DB08931	Riociguat	GUCY1A1

DB08931	Riociguat	GUCY1A2
DB08931	Riociguat	GUCY1B1
DB08932	Macitentan	EDNRA
DB08932	Macitentan	EDNRB
DB08941	Isoxsuprine	ADRB2
DB09026	Aliskiren	REN
DB09029	Secukinumab	IL17A
DB09033	Vedolizumab	ITGA4
DB09033	Vedolizumab	ITGB7
DB09034	Suvorexant	HCRTR1
DB09034	Suvorexant	HCRTR2
DB09035	Nivolumab	PDCD1
DB09036	Siltuximab	IL6
DB09037	Pembrolizumab	PDCD1
DB09039	Eliglustat	UGCG
DB09046	Metreleptin	LEPR
DB09051	Elosulfase alfa	GALNS
DB09053	Ibrutinib	BTK
DB09061	Cannabidiol	CNR1
DB09061	Cannabidiol	CNR2
DB09067	Corticorelin ovine triflutate	CRHR1
DB09067	Corticorelin ovine triflutate	CRHR2
DB09071	Tasimelteon	MTNR1A
DB09071	Tasimelteon	MTNR1B
DB09074	Olaparib	PARP1
DB09074	Olaparib	PARP2
DB09074	Olaparib	PARP3
DB09075	Edoxaban	F10
DB09079	Nintedanib	FGFR1
DB09079	Nintedanib	FGFR2
DB09079	Nintedanib	FGFR3
DB09079	Nintedanib	FLT1
DB09079	Nintedanib	FLT4
DB09079	Nintedanib	KDR
DB09079	Nintedanib	PDGFRA
DB09079	Nintedanib	PDGFRB
DB09089	Trimebutine	OPRK1
DB09089	Trimebutine	OPRM1
DB09099	Somatostatin	SSTR1
DB09099	Somatostatin	SSTR2
DB09099	Somatostatin	SSTR3
DB09099	Somatostatin	SSTR4
DB09099	Somatostatin	SSTR5
DB09105	Asfotase alfa	ALPL
DB09122	Peginterferon beta-1a	IFNAR1
DB09122	Peginterferon beta-1a	IFNAR2
DB09211	Limaprost	PTGER1
DB09212	Loxoprofen	PTGS1
DB09212	Loxoprofen	PTGS2
DB09224	Melperone	DRD2
DB09228	Conestat alfa	C1R
DB09228	Conestat alfa	C1S
DB09228	Conestat alfa	KLKB1
DB09230	Azelnidipine	CACNA1C
DB09230	Azelnidipine	CACNA1D
DB09230	Azelnidipine	CACNA1F
DB09230	Azelnidipine	CACNA1S
DB09232	Cilnidipine	CACNA1B
DB09232	Cilnidipine	CACNA1C
DB09232	Cilnidipine	CACNA1D
DB09232	Cilnidipine	CACNA1F
DB09232	Cilnidipine	CACNA1S
DB09260	Parnaparin	SERPINC1
DB09265	Lixisenatide	GLP1R
DB09280	Lumacaftor	CFTR
DB09292	Sacubitril	MME
DB09302	Alirocumab	PCSK9
DB09303	Evolocumab	PCSK9
DB09331	Daratumumab	CD38
DB09345	Pramocaine	SCN1A
DB09345	Pramocaine	SCN2A
DB09345	Pramocaine	SCN3A
DB09345	Pramocaine	SCN5A
DB09345	Pramocaine	SCN9A
DB09539	Omega-3-acid ethyl esters	CETP
DB09539	Omega-3-acid ethyl esters	LPL
DB09570	Ixazomib	PSMB5
DB11362	Selexipag	PTGIR
DB11526	Masitinib	KIT
DB11563	Sebelipase alfa	LIPA
DB11569	Ixekizumab	IL17A
DB11580	Ravulizumab	C5
DB11581	Venetoclax	BCL2
DB11595	Atezolizumab	CD274
DB11614	Rupatadine	HRH1
DB11614	Rupatadine	PTAFR
DB11632	Opicapone	COMT
DB11636	Nomegestrol	PGR
DB11640	Amifampridine	KCNA4
DB11640	Amifampridine	KCND2
DB11642	Pitolisant	HRH3
DB11644	Tafamidis	TTR
DB11657	Tabalumab	TNFSF13B
DB11675	Mosapride	HTR4
DB11689	Selumetinib	MAP2K1
DB11689	Selumetinib	MAP2K2

DB11693	Voclosporin	PPP3CA
DB11693	Voclosporin	PPP3CB
DB11693	Voclosporin	PPP3CC
DB11693	Voclosporin	PPP3R1
DB11693	Voclosporin	PPP3R2
DB11703	Acalabrutinib	BTK
DB11708	Peficitinib	JAK1
DB11708	Peficitinib	JAK2
DB11708	Peficitinib	JAK3
DB11712	Tezacaftor	CFTR
DB11714	Durvalumab	CD274
DB11725	Latrepirdine	ACHE
DB11725	Latrepirdine	GRIN1
DB11725	Latrepirdine	GRIN2A
DB11725	Latrepirdine	GRIN2B
DB11725	Latrepirdine	GRIN2C
DB11725	Latrepirdine	GRIN2D
DB11737	Icotinib	EGFR
DB11738	Rilmenidine	ADRA2A
DB11738	Rilmenidine	ADRA2B
DB11738	Rilmenidine	ADRA2C
DB11750	Clobetasol	NR3C1
DB11757	Istradefylline	ADORA2A
DB11758	Cenicriviroc	CCR2
DB11758	Cenicriviroc	CCR5
DB11767	Sarilumab	IL6R
DB11771	Tremelimumab	CTLA4
DB11776	Brodalumab	IL17RA
DB11783	Imidapril	ACE
DB11803	Sirukumab	IL6
DB11805	Saracatinib	ABL1
DB11805	Saracatinib	SRC
DB11817	Baricitinib	JAK1
DB11817	Baricitinib	JAK2
DB11823	Esketamine	GRIN1
DB11823	Esketamine	GRIN2A
DB11823	Esketamine	GRIN2B
DB11823	Esketamine	GRIN2C
DB11823	Esketamine	GRIN2D
DB11834	Guselkumab	IL23A
DB11837	Osilodrostat	CYP11B2
DB11842	Angiotensin II	AGTR1
DB11842	Angiotensin II	AGTR2
DB11850	Ublituximab	MS4A1
DB11859	Brexanolone	GABRA1
DB11859	Brexanolone	GABRA2
DB11859	Brexanolone	GABRA3
DB11859	Brexanolone	GABRA4
DB11859	Brexanolone	GABRA5
DB11859	Brexanolone	GABRA6
DB11859	Brexanolone	GABRB1
DB11859	Brexanolone	GABRB2
DB11859	Brexanolone	GABRB3
DB11859	Brexanolone	GABRD
DB11859	Brexanolone	GABRE
DB11859	Brexanolone	GABRG1
DB11859	Brexanolone	GABRG2
DB11859	Brexanolone	GABRG3
DB11859	Brexanolone	GABRP
DB11859	Brexanolone	GABRQ
DB11864	Preladenant	ADORA2A
DB11914	Lebrikizumab	IL13
DB11915	Valbenazine	SLC18A2
DB11921	Deflazacort	NR3C1
DB11947	Pridopidine	DRD2
DB11967	Binimetinib	MAP2K1
DB11967	Binimetinib	MAP2K2
DB11976	Anifrolumab	IFNAR1
DB11976	Anifrolumab	IFNAR2
DB11988	Ocrelizumab	MS4A1
DB12010	Fostamatinib	SYK
DB12014	Ozoralizumab	TNF
DB12016	Ponesimod	S1PR1
DB12023	Benralizumab	IL5RA
DB12053	Visilizumab	CD3D
DB12053	Visilizumab	CD3E
DB12053	Visilizumab	CD3G
DB12061	Pardoprunox	DRD2
DB12061	Pardoprunox	DRD3
DB12061	Pardoprunox	HTR1A
DB12118	Sotatercept	ACVR2A
DB12119	Gevokizumab	IL1B
DB12146	Rigosertib	PLK1
DB12154	Itacitinib	JAK1
DB12159	Dupilumab	IL4R
DB12169	Tralokinumab	IL13
DB12189	Etrolizumab	ITGB7
DB12193	Ajulemic acid	CNR2
DB12203	Tozadenant	ADORA2A
DB12233	Iguratimod	NFKB1
DB12235	Estetrol	ESR1
DB12235	Estetrol	ESR2
DB12270	Losmapimod	MAPK11
DB12270	Losmapimod	MAPK12
DB12270	Losmapimod	MAPK13
DB12270	Losmapimod	MAPK14

DB12278	Propiverine	CACNA1C
DB12278	Propiverine	CACNA1D
DB12278	Propiverine	CACNA1F
DB12278	Propiverine	CACNA1S
DB12278	Propiverine	CHRM1
DB12278	Propiverine	CHRM2
DB12278	Propiverine	CHRM3
DB12278	Propiverine	CHRM4
DB12278	Propiverine	CHRM5
DB12294	Anrukinzumab	IL13
DB12299	Bavisant	HRH3
DB12321	Ifetroban	TBXA2R
DB12335	Tanezumab	NGF
DB12366	Vestronidase alfa	GUSB
DB12369	Sotrastaurin	PRKCD
DB12369	Sotrastaurin	PRKCE
DB12369	Sotrastaurin	PRKCH
DB12369	Sotrastaurin	PRKCQ
DB12371	Siponimod	S1PR1
DB12371	Siponimod	S1PR5
DB12374	Velmanase alfa	MAN2B1
DB12396	Fresolimumab	TGFB1
DB12396	Fresolimumab	TGFB2
DB12396	Fresolimumab	TGFB3
DB12421	Somavaratan	GHR
DB12435	Tipelukast	CYSLTR1
DB12440	Verdiperstat	MPO
DB12462	Ralinepag	PTGIR
DB12478	Piribedil	DRD2
DB12478	Piribedil	DRD3
DB12498	Mogamulizumab	CCR4
DB12525	Molgramostim	CSF2RA
DB12534	Mavrilimumab	CSF2RA
DB12555	Nelotanserin	HTR2A
DB12584	Bimagrumab	ACVR2A
DB12584	Bimagrumab	ACVR2B
DB12612	Ozanimod	S1PR1
DB12612	Ozanimod	S1PR2
DB12612	Ozanimod	S1PR3
DB12612	Ozanimod	S1PR4
DB12612	Ozanimod	S1PR5
DB12614	Reparixin	CXCR1
DB12614	Reparixin	CXCR2
DB12617	Mizoribine	IMPDH1
DB12617	Mizoribine	IMPDH2
DB12656	Somatorelin	GHRHR
DB12705	Cenerimod	S1PR1
DB12732	Firategrast	ITGA4
DB12773	Sifalimumab	IFNA1
DB12773	Sifalimumab	IFNA10
DB12773	Sifalimumab	IFNA13
DB12773	Sifalimumab	IFNA14
DB12773	Sifalimumab	IFNA16
DB12773	Sifalimumab	IFNA17
DB12773	Sifalimumab	IFNA2
DB12773	Sifalimumab	IFNA21
DB12773	Sifalimumab	IFNA4
DB12773	Sifalimumab	IFNA5
DB12773	Sifalimumab	IFNA6
DB12773	Sifalimumab	IFNA7
DB12773	Sifalimumab	IFNA8
DB12783	Benserazide	DDC
DB12844	Rontalizumab	IFNA1
DB12844	Rontalizumab	IFNA10
DB12844	Rontalizumab	IFNA13
DB12844	Rontalizumab	IFNA14
DB12844	Rontalizumab	IFNA16
DB12844	Rontalizumab	IFNA17
DB12844	Rontalizumab	IFNA2
DB12844	Rontalizumab	IFNA21
DB12844	Rontalizumab	IFNA4
DB12844	Rontalizumab	IFNA5
DB12844	Rontalizumab	IFNA6
DB12844	Rontalizumab	IFNA7
DB12844	Rontalizumab	IFNA8
DB12863	Sivelestat	ELANE
DB12877	Oxatomide	ALOX5
DB12891	Ozanezumab	RTN4
DB12917	Bimekizumab	IL17A
DB12917	Bimekizumab	IL17F
DB12943	Milatuzumab	CD74
DB12973	Serlopitant	TACR1
DB13036	Ramatroban	PTGDR
DB13036	Ramatroban	TBXA2R
DB13074	Macimorelin	GHSR
DB13139	Levosalbutamol	ADRB2
DB13144	Lenograstim	CSF3R
DB13161	Nusinersen	SMN2
DB13169	Nandrolone	AR
DB13173	Cerliponase alfa	TPP1
DB13399	Terguride	DRD2
DB13399	Terguride	PRL
DB13679	Dexchlorpheniramine	HRH1
DB13688	Drisapersen	DMD
DB13728	Halometasone	NR3C1
DB13867	Fluticasone	NR3C1

DB13919	Candesartan	AGTR1
DB13926	Cenegermin	NGFR
DB13928	Semaglutide	GLP1R
DB13944	Testosterone enanthate	AR
DB13946	Testosterone undecanoate	AR
DB13981	Nomegestrol acetate	PGR
DB14004	Tildrakizumab	IL23A
DB14218	Telotristat	TPH1
DB14218	Telotristat	TPH2
DB14512	Mometasone furoate	NR3C1
DB14539	Hydrocortisone acetate	NR3C1
DB14541	Hydrocortisone cypionate	NR3C1
DB14545	Hydrocortisone succinate	NR3C1
DB14644	Methylprednisolone hemisuccinate	NR3C1
DB14646	Prednisone acetate	NR3C1
DB14681	Cortisone	NR3C1
DB14712	Elapegademase	ADA
DB14713	Inotersen	TTR
DB14724	Emapalumab	IFNG
DB14726	Dabigatran	F2
DB14751	Mecasermin rinfabate	GHR
DB14751	Mecasermin rinfabate	IGF1R
DB14762	Risankizumab	IL23A
DB14766	Etrasimod	S1PR1
DB14766	Etrasimod	S1PR2
DB14766	Etrasimod	S1PR3
DB14766	Etrasimod	S1PR4
DB14766	Etrasimod	S1PR5
DB14845	Filgotinib	JAK1
DB14867	Parsaclisib	PIK3CD
DB14872	Lucerastat	UGCG
DB14899	Tavapadon	DRD1
DB14899	Tavapadon	DRD5
DB14910	Mirikizumab	IL23A
DB14916	Selonsertib	MAP3K5
DB14952	Pamrevlumab	CCN2
DB14966	Venglustat	UGCG
DB14976	Relacorilant	NR3C1
DB14991	Nomacopan	C5
DB14996	Sutimlimab	C1S
DB14997	Pateclizumab	LTA
DB15005	Viltolarsen	DMD
DB15011	Avacopan	C5AR1
DB15019	Mongersen	SMAD7
DB15067	Volanesorsen	APOC3
DB15083	Domagrozumab	MSTN
DB15091	Upadacitinib	JAK1
DB15093	Somapacitan	GHR
DB15097	Gefapixant	P2RX3
DB15114	Vamorolone	NR3C1
DB15130	Dexpramipexole	DRD1
DB15130	Dexpramipexole	DRD2
DB15130	Dexpramipexole	DRD3
DB15130	Dexpramipexole	DRD4
DB15130	Dexpramipexole	DRD5
DB15155	Pepinemab	SEMA4D
DB15240	Elezanumab	RGMA
DB15253	Bleselumab	CD40
DB15256	Reldesemtiv	TNNC2
DB15260	Iscalimab	CD40
DB15305	Risdiplam	SMN2
DB15347	Branebrutinib	BTK
DB15444	Elexacaftor	CFTR

Supplemental Table 3. Number of clinical trials, diseases, drugs and drug target genes in the four clinical trial registries.

Registries	Clinical trials	Disease groups	Drugs	Target genes
**JPRN**	621	88	201	269
**ClinicalTrials.gov**	5,403	174	677	522
**EUCTR**	2,075	123	415	405
**ChiCTR**	208	49	96	154
**Total ***	8,307	189	753	551

* Non-redundant numbers for disease groups, drugs and target genes.

Supplemental Table 4. Top 100 R_{gene} disease pairs.

* 1. No drug repositioning events, 2. No Phase 3 nor Phase 4 clinical trials

	Disease A		Disease B			
Rank	ID	Disease name	ID	Disease name	R_{gene}	Note *
1	13	Multiple sclerosis	96	Crohn disease	107.78	
2	13	Multiple sclerosis	46	Malignant rheumatoid arthritis	103.88	
3	2	Amyotrophic lateral sclerosis	13	Multiple sclerosis	99.64	
4	46	Malignant rheumatoid arthritis	96	Crohn disease	92.11	
5	6	Parkinson disease	13	Multiple sclerosis	82.27	
6	46	Malignant rheumatoid arthritis	97	Ulcerative colitis	75.84	
7	96	Crohn disease	97	Ulcerative colitis	75.07	
8	2	Amyotrophic lateral sclerosis	96	Crohn disease	72.50	
9	2	Amyotrophic lateral sclerosis	6	Parkinson disease	69.91	
10	13	Multiple sclerosis	97	Ulcerative colitis	66.94	
11	46	Malignant rheumatoid arthritis	49	Systemic lupus erythematosus	65.60	
12	2	Amyotrophic lateral sclerosis	46	Malignant rheumatoid arthritis	64.70	
13	6	Parkinson disease	46	Malignant rheumatoid arthritis	60.95	
14	6	Parkinson disease	8	Huntington disease	60.84	2
15	13	Multiple sclerosis	156	Rett syndrome	60.74	
16	2	Amyotrophic lateral sclerosis	8	Huntington disease	55.20	
17	2	Amyotrophic lateral sclerosis	156	Rett syndrome	53.58	
18	5	Progressive supranuclear palsy	6	Parkinson disease	52.55	
19	6	Parkinson disease	96	Crohn disease	52.39	
20	8	Huntington disease	13	Multiple sclerosis	50.69	
21	13	Multiple sclerosis	49	Systemic lupus erythematosus	48.93	
22	2	Amyotrophic lateral sclerosis	5	Progressive supranuclear palsy	47.82	
23	6	Parkinson disease	17	Multiple system atrophy	47.33	
24	96	Crohn disease	156	Rett syndrome	46.20	2
25	6	Parkinson disease	70	Spinal stenosis	44.74	
26	46	Malignant rheumatoid arthritis	51	Systemic scleroderma	44.61	
27	46	Malignant rheumatoid arthritis	84	Sarcoidosis	44.34	
28	140	Dorabe syndrome	144	Lennox-Gastaut syndrome	42.00	
29	13	Multiple sclerosis	70	Spinal stenosis	41.09	
30	6	Parkinson disease	97	Ulcerative colitis	40.82	
31	6	Parkinson disease	140	Dorabe syndrome	40.27	2
32	46	Malignant rheumatoid arthritis	70	Spinal stenosis	39.98	
33	51	Systemic scleroderma	85	Idiopathic interstitial pneumonia	39.85	
34	5	Progressive supranuclear palsy	8	Huntington disease	39.85	2
35	2	Amyotrophic lateral sclerosis	17	Multiple system atrophy	39.66	2
36	5	Progressive supranuclear palsy	13	Multiple sclerosis	39.52	
37	34	Neurofibromatosis	46	Malignant rheumatoid arthritis	39.47	
38	6	Parkinson disease	193	Prader-Willi syndrome	39.14	2
38	6	Parkinson disease	144	Lennox-Gastaut syndrome	39.14	
40	13	Multiple sclerosis	17	Multiple system atrophy	39.01	
41	3	Spinal muscular atrophy	5	Progressive supranuclear palsy	38.96	1,2
42	6	Parkinson disease	206	Fragile X syndrome	38.92	
43	13	Multiple sclerosis	206	Fragile X syndrome	38.74	
44	8	Huntington disease	46	Malignant rheumatoid arthritis	38.67	2
45	2	Amyotrophic lateral sclerosis	97	Ulcerative colitis	38.42	2
46	6	Parkinson disease	231	Alpha-1-antitrypsin deficiency	37.90	2
47	6	Parkinson disease	298	Hereditary pancreatitis	37.68	
48	2	Amyotrophic lateral sclerosis	3	Spinal muscular atrophy	37.57	

49	13	Multiple sclerosis	86	Pulmonary arterial hypertension	37.53	
50	5	Progressive supranuclear palsy	17	Multiple system atrophy	37.49	2
51	140	Dorabe syndrome	193	Prader-Willi syndrome	37.19	2
52	46	Malignant rheumatoid arthritis	299	Cystic fibrosis	36.91	
53	6	Parkinson disease	156	Rett syndrome	36.39	
54	140	Dorabe syndrome	156	Rett syndrome	36.37	1,2
55	144	Lennox-Gastaut syndrome	193	Prader-Willi syndrome	36.08	2
56	2	Amyotrophic lateral sclerosis	144	Lennox-Gastaut syndrome	36.01	2
57	46	Malignant rheumatoid arthritis	271	Ankylosing spondylitis	36.00	
58	6	Parkinson disease	296	Biliary atresia	35.00	2
59	49	Systemic lupus erythematosus	96	Crohn disease	34.58	
60	13	Multiple sclerosis	231	Alpha-1-antitrypsin deficiency	34.36	
61	84	Sarcoidosis	96	Crohn disease	34.27	
61	6	Parkinson disease	84	Sarcoidosis	34.27	
63	70	Spinal stenosis	96	Crohn disease	34.24	
64	144	Lennox-Gastaut syndrome	156	Rett syndrome	34.17	1,2
65	46	Malignant rheumatoid arthritis	50	Dermatomyositis	33.87	
66	8	Huntington disease	206	Fragile X syndrome	33.62	2
67	8	Huntington disease	17	Multiple system atrophy	33.56	1,2
68	46	Malignant rheumatoid arthritis	53	Sjogren syndrome	33.39	
69	13	Multiple sclerosis	299	Cystic fibrosis	33.21	
70	3	Spinal muscular atrophy	8	Huntington disease	33.20	2
71	13	Multiple sclerosis	53	Sjogren syndrome	33.13	
72	13	Multiple sclerosis	113	Muscular dystrophy	33.10	
73	156	Rett syndrome	206	Fragile X syndrome	32.87	2
74	46	Malignant rheumatoid arthritis	298	Hereditary pancreatitis	32.85	
75	13	Multiple sclerosis	84	Sarcoidosis	32.79	
76	3	Spinal muscular atrophy	13	Multiple sclerosis	32.48	
77	97	Ulcerative colitis	298	Hereditary pancreatitis	32.24	
78	6	Parkinson disease	86	Pulmonary arterial hypertension	32.08	
79	2	Amyotrophic lateral sclerosis	140	Dorabe syndrome	32.01	2
80	13	Multiple sclerosis	140	Dorabe syndrome	31.71	2
81	46	Malignant rheumatoid arthritis	86	Pulmonary arterial hypertension	31.65	2
82	156	Rett syndrome	193	Prader-Willi syndrome	31.43	1,2
83	96	Crohn disease	298	Hereditary pancreatitis	31.42	
84	46	Malignant rheumatoid arthritis	56	Behcet disease	31.21	
85	11	Myasthenia gravis	13	Multiple sclerosis	31.17	
86	2	Amyotrophic lateral sclerosis	34	Neurofibromatosis	31.14	2
87	13	Multiple sclerosis	298	Hereditary pancreatitis	31.00	
88	13	Multiple sclerosis	193	Prader-Willi syndrome	30.97	2
88	13	Multiple sclerosis	144	Lennox-Gastaut syndrome	30.97	
90	2	Amyotrophic lateral sclerosis	206	Fragile X syndrome	30.92	2
91	8	Huntington disease	156	Rett syndrome	30.92	
92	84	Sarcoidosis	97	Ulcerative colitis	30.86	
93	46	Malignant rheumatoid arthritis	113	Muscular dystrophy	30.84	
94	6	Parkinson disease	226	Interstitial cystitis with Hunners ulcer	30.79	
95	70	Spinal stenosis	298	Hereditary pancreatitis	30.52	
96	8	Huntington disease	144	Lennox-Gastaut syndrome	30.40	1,2
97	13	Multiple sclerosis	226	Interstitial cystitis with Hunners ulcer	30.12	
98	86	Pulmonary arterial hypertension	226	Interstitial cystitis with Hunners ulcer	30.09	
99	70	Spinal stenosis	97	Ulcerative colitis	30.08	
100	8	Huntington disease	140	Dorabe syndrome	30.05	1,2

Supplemental Table 5. 33 diseases appeared in the top 100 R_{gene} disease pairs.

* Description of disease names are simplified for search. (e.g. Parkinson's disease -> Parkinson disease)

ID	Disease name *
2	Amyotrophic lateral sclerosis
3	Spinal muscular atrophy
5	Progressive supranuclear palsy
6	Parkinson disease
8	Huntington disease
11	Myasthenia gravis
13	Multiple sclerosis
17	Multiple system atrophy
34	Neurofibromatosis
46	Malignant rheumatoid arthritis
49	Systemic lupus erythematosus
50	Dermatomyositis
51	Systemic scleroderma
53	Sjogren syndrome
56	Behcet disease
70	Spinal stenosis
84	Sarcoidosis
85	Idiopathic interstitial pneumonia
86	Pulmonary arterial hypertension
96	Crohn disease
97	Ulcerative colitis
113	Muscular dystrophy
140	Dorabe syndrome
144	Lennox-Gastaut syndrome
156	Rett syndrome
193	Prader-Willi syndrome
206	Fragile X syndrome
226	Interstitial cystitis with Hunners ulcer
231	Alpha-1-antitrypsin deficiency
271	Ankylosing spondylitis
296	Biliary atresia
298	Hereditary pancreatitis
299	Cystic fibrosis
